# The association between different aspects of socioeconomic deprivation and severe maternal morbidity

**DOI:** 10.1111/aogs.70134

**Published:** 2025-12-29

**Authors:** Dorothea Geddes‐Barton, Rema Ramakrishnan, Raph Goldacre, Marian Knight

**Affiliations:** ^1^ National Perinatal Epidemiology Unit, Nuffield Department of Population Health University of Oxford Oxford UK; ^2^ Oxford NIHR Biomedical Research Centre Oxford UK; ^3^ Nuffield Department of Population Health Oxford University Oxford UK

**Keywords:** health inequalities, maternal health, severe maternal morbidity, socioeconomic disadvantage

## Abstract

**Introduction:**

Living in a deprived neighborhood is associated with an increased risk of severe maternal morbidity (SMM), but the specific deprivation factors or individual SMM conditions driving this risk remain unclear. This study examined how different domains and subdomains of the Index of Multiple Deprivation (IMD) are associated with SMM, identifying key contributors.

**Material and Methods:**

We conducted a nationwide, population‐based cohort study using English Hospital Episode Statistics Admitted Patient Care (HES APC) data. The cohort included 4 040 106 women aged 10–55 years who gave birth in NHS facilities in England between January 1, 2013, and March 31, 2023, with pregnancies of ≥20 weeks' gestation. Multilevel multivariable Poisson regression estimated adjusted risk ratios (aRR) and 95% confidence intervals (CI) of composite SMM and key individual SMM conditions for each IMD quintile compared to the least deprived quintile, and aRR (95% CI) of composite SMM in each IMD domain/subdomain quintile compared to the least deprived quintile.

**Results:**

IMD domains showed varying associations with SMM. Income and employment deprivation had the strongest associations, with women living in the most deprived quintile having aRRs of 1.16 (95% CI 1.12–1.20) and 1.15 (95% CI 1.11–1.19) compared to those living in the least deprived quintile, respectively. Contrastingly, high geographical barriers to services were associated with a lower risk of SMM (aRR: 0.92 (95% CI 0.88–0.95)). Sepsis, acute cardiac events, and embolism play a key role in the association between composite deprivation and SMM, with women living in the most deprived areas having risk ratios of 1.43 (95% CI 1.36–1.50), 1.24 (95% CI 1.09–1.41), and 1.97 (95% CI 1.69–2.29), respectively, for each of the conditions, compared to women living in the least deprived areas.

**Conclusions:**

There appears to be a widening gap in the risk of SMM between women living in the least and most deprived areas in England, with sepsis, cardiac events, and embolism having the strongest association with deprivation. Composite measures of area‐level deprivation may obscure the diverse impacts of specific deprivation factors, and individual‐level socioeconomic measures are needed to clarify pathways contributing to SMM risk.

## Graphical abstract

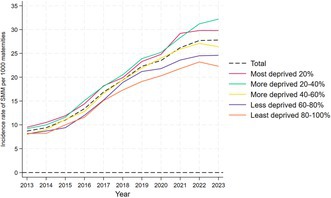

**Abbreviations**
aRR: adjusted risk ratio
CI: confidence interval
HES APC: Hospital Episode Statistics Admitted Patient Care
IMD: Index of Multiple Deprivation
NHS: National Health Service
PAF: population attributable fraction
SMM: severe maternal morbidity

**Key message**

Socioeconomic disadvantage is associated with severe maternal morbidity (SMM), and the gap appears to be widening. Sepsis, embolism, and cardiac events are key conditions driving the association. However, more precise measures of disadvantage are needed to understand the causal pathways to SMM.

## 1 INTRODUCTION

In England, women living in the most deprived areas have more than twice the risk of maternal death compared to those living in the least deprived areas, and this gap is widening.^1^ However, for every maternal death, many more women experience serious health complications around the time of childbirth. These complications, collectively referred to as severe maternal morbidity (SMM), occur approximately 100 times more frequently than maternal mortality. A population‐based study using data from 2016 to 2021 identified a clear dose–response relationship where the odds of experiencing SMM increased with greater levels of deprivation.^2^ Yet, it remains unclear which specific SMM conditions are driving this trend, complicating efforts to identify causal pathways.

In addition, this study,^3^ alongside many other population‐based SMM studies in high‐income countries, relies on neighborhood‐level composite deprivation measures, because of their availability in administrative data.^4^ These measures combine a range of indicators, including income, employment, housing, education, and environmental conditions, to provide a broad measure of socioeconomic disadvantage.^4^ While these composite measures offer a useful overview of deprivation and capture both individual and contextual factors, when used as a marker of socioeconomic disadvantage in health research, they can obscure important heterogeneity within populations and make interpretation more challenging.^5^ As a result, the distinct relationships between specific dimensions of deprivation, such as education, housing, or income, and SMM remain poorly understood.

Disaggregated analyses are particularly important for minoritized ethnic groups, who are both overrepresented in the most deprived areas^2^, ^6^ and face higher risks of maternal mortality and SMM.^1^, ^2^, ^6^ While a recent study in England found limited evidence for effect modification by ethnicity in the association between overall deprivation and SMM,^2^ it remains unclear whether these findings hold when examining specific domains of deprivation, such as education or housing, in relation to SMM outcomes.

To address these gaps, this study disaggregated both the composite neighborhood deprivation measure, the Index of Multiple Deprivation (IMD) and the composite SMM outcome into their component parts to explore the specific associations between deprivation and SMM. It also examined whether these relationships varied across ethnic groups, providing insights that can guide more precise and impactful responses to maternal health inequities.

## 2 MATERIAL AND METHODS

### 2.1 Study design

A retrospective nationwide population‐based cohort study was conducted using the English National Hospital Episode Statistics Admitted Patient Care (HES APC) database. Patient and public involvement for this study is described in Box 1.

BOX 1 **Patient and public involvement.**

We conducted a focus group with six women who had a recent experience with pregnancy and faced multiple disadvantages. The discussion focused on using routine health data to assess neighborhood deprivation. The women were comfortable with having this data collected and using it to categorize the level of disadvantage. They expressed support for using routine data to enhance understanding of how these factors impact pregnancy outcomes.

The participants emphasized the critical role of ethnicity in maternal health research, particularly how it relates to socioeconomic disadvantage, prompting a modification of the study design to explore how different aspects of deprivation intersect with ethnicity.

The group also shared personal experiences of socioeconomic disadvantage, highlighting the detrimental effects of poor housing and housing insecurity on pregnancy, as well as the challenges of managing unemployment benefits. They discussed how these factors negatively impacted their pregnancies. Additionally, they spoke of experiences of discrimination and disrespect from maternity staff due to their social factors. Despite these challenges, they expressed a desire for their sociodemographic information, such as income level and educational attainment, to be shared with healthcare providers, believing that this would better support the care given to women during pregnancy.

### 2.2 Data source

The HES APC, a national database that records hospital admissions within the National Health Service (NHS), covers 97% of births in England.^7^ It includes demographic and clinical data and specific maternity information. More information about the HES APC and the data cleaning process is available elsewhere.^8^ In the HES APC, the diagnoses are based on the International Classification of Diseases, 10th revision (ICD‐10) codes, and operative procedures follow the Office of Population Censuses and Surveys Classification of Interventions and Procedures, version 4 (OPCS‐4) classifications.

### 2.3 Ethics committee approval, data availability and reporting

Under the assessment of the NHS Health Research Authority, using the HES APC data to conduct epidemiological and health service research at the University of Oxford does not need research ethics committee approval as it is anonymized data. This study is reported according to the RECORD Guidelines.^9^

### 2.4 Study population, exposure and outcome

This study included all women aged 10–55 years who gave birth (singleton and multiple pregnancies) between 1st January 1, 2013, and March 31, 2023, in a hospital and with a gestational age ≥20 weeks. Women with missing IMD data (2.3%) were excluded. If a woman had more than one pregnancy and birth during this period, only one birth was randomly selected to avoid clustering effects, as the multilevel models chosen failed to converge when they included two levels (IMD and multiple births per woman). The primary exposure was the composite IMD, and the secondary exposures were nine domains and subdomains. Further information on the IMD is detailed in the supporting information Methods. The Health Deprivation and Disability domain was excluded because its rankings are based on health outcome data from HES APC. National rankings were categorized into quintiles, with the first being the least deprived and the fifth most deprived. Outcomes included SMM at the time of birth, identified using an updated English Maternal Morbidity Outcome Indicator (EMMOI), which includes 21 diagnoses and 16 procedures.^10^, ^11^ The details of which are shown in Table S1.

Covariates were selected using a Directed Acyclic Graph (DAG). Information on the covariates is included in the supporting information Methods and Table S2.

### 2.5 Statistical analysis

Statistical analysis was performed using Stata version 18 (StataCorp. 2023. Stata Statistical Software: Release 18. College Station, TX: StataCorp LLC). Statistical significance was assumed to be a *p*‐value <0.05. A complete case analysis excluding 200 950 women with missing information on ethnicity was used for all models.

The characteristics of included women are presented as number and percentage stratified by the composite IMD quintile. The incidence rate of SMM for each composite IMD quintile and each ethnic group was calculated as SMM per 1000 maternities. Pearson correlation coefficients were calculated between the different domain/subdomains of the IMD. A multilevel Poisson regression model with a random intercept was used to account for clustering between individuals who shared the same postcode (LSOA).^8^ For the composite IMD and each domain/subdomain of IMD, multilevel multivariable Poisson regression models were built to estimate the risk ratio (95% confidence interval) of SMM in the different quintiles of deprivation compared to the least deprived quintile, respectively. Model 1 adjusted for age, year of birth, parity, and ethnicity. Model 2 included model 1 and any additional variables identified as confounders for that specific domain/subdomain (which did not apply to the composite IMD measure). Model 3 included model 2 and pre‐existing medical conditions, mental health, and smoking/substance misuse. Additionally, Model 1 was used to compute estimates separately for the major components of the composite SMM outcome.

### 2.6 Effect modification by ethnicity

Likelihood ratio testing evaluated effect modification by ethnicity. If P_interaction_ <0.05 indicated effect modification, all ethnic groups were compared to a reference point (the least deprived White women category). Absolute measures were calculated using average adjusted predictive margins to compute average probabilities of SMM for each ethnicity and IMD quintile category.

### 2.7 Population attributable fraction (PAFs)

Univariable and multivariable population attributable fractions (PAFs) and their 95% confidence intervals were calculated using the “punaf” command in Stata for each measure of socioeconomic deprivation. The attributable fraction was defined as the difference between observed and expected SMM cases, divided by the observed number of SMM cases. This fraction indicates the proportion of SMM that would not have occurred if the risk matched that of the reference group. The analysis used women in the highest socioeconomic quintile or those from a White ethnic background living in the highest socioeconomic quintile as the reference group, applied to the entire cohort.

## 3 RESULTS

### 3.1 Descriptive statistics

**FIGURE 1:**
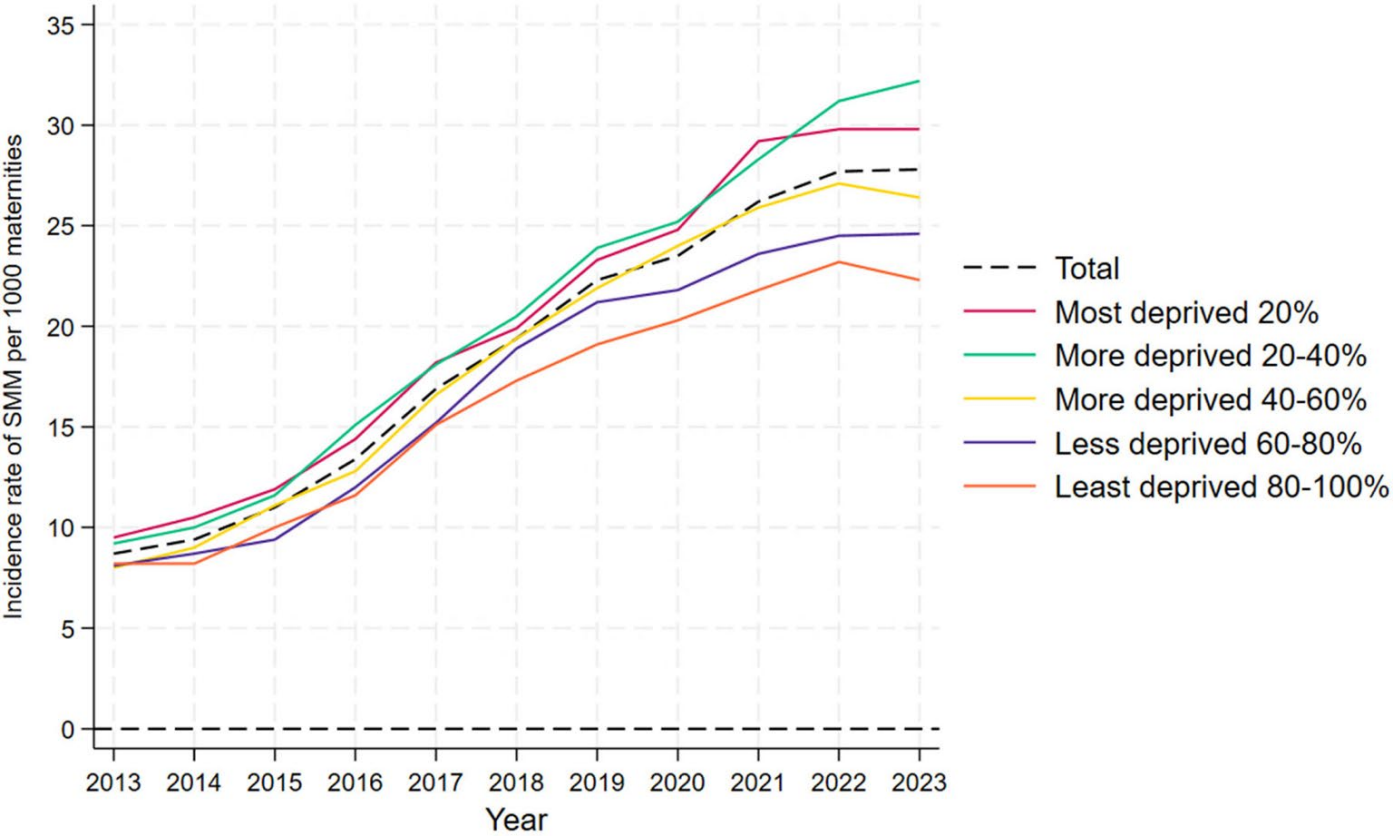
The crude incidence rate (*N* per 1000 maternities) of SMM over time by the composite Index of Multiple Deprivation.

The study included 4 040 106 women with a mean age of 30 at childbirth. Details of the cohort are shown in Figure S1 and Table S3. The overall incidence of SMM was 17.7 (95% CI 17.6–17.8) per 1000 maternities. Women living in the most deprived quintile were more likely to experience SMM, be younger, belong to minoritized ethnic groups, live in urban areas with higher percentages of non‐UK born residents, and reside in Yorkshire and the Humber, the North West, the West Midlands, or London. They were also more likely to be multiparous, have a history of smoking, or substance misuse, be obese, have pre‐existing mental health issues, have unassisted vertex births and register their pregnancies ≥12 weeks. During 2013–2023, the incidence rate of SMM appeared to increase over time with a gap between the most and least deprived IMD quintiles (Figure 1). This increase over time was also evident among different ethnic groups (Figures S2 and S3). The main causes of SMM were sepsis (40%), acute renal failure (14.4%), and management of intra‐abdominal or vaginal collections (14.7%) (Figure S4). Sepsis and acute renal failure appeared to be the main conditions driving the increase over time. Income and employment were the most closely correlated domains/subdomains (correlation coefficient: 0.901) (Table S4).

### 3.2 Multivariable analysis

**FIGURE 2:**
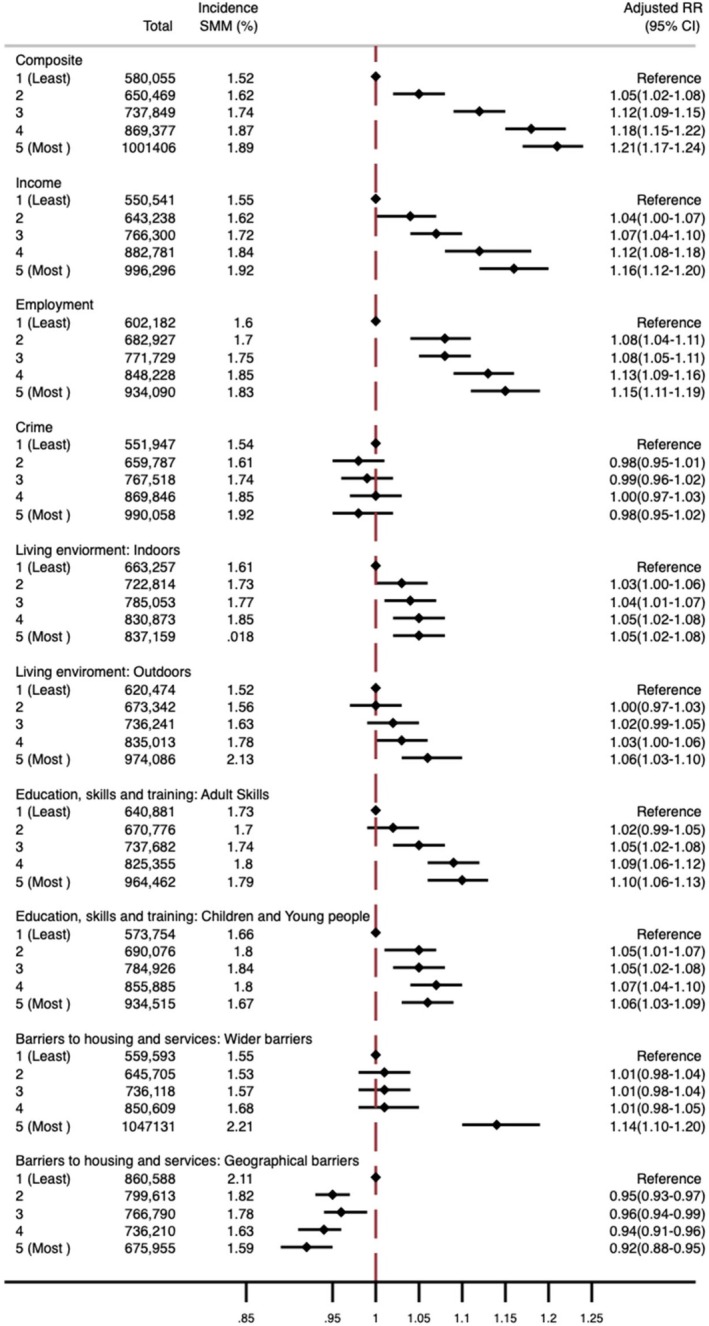
Adjusted risk ratio and 95% confidence interval (RR [95% CI]) of severe maternal morbidity (SMM) in each of the composite IMD quintiles compared to the least deprived quintile. All domains are adjusted for age, year, parity, and ethnicity. Income and employment are additionally adjusted for neighborhood % not born in the United Kingdom and neighborhood English language proficiency. Indoor living environment is additionally adjusted for neighborhood income and neighborhood English language proficiency. The outdoor living environment is additionally adjusted for neighborhood income, neighborhood English language proficiency and neighborhood % not born in the United Kingdom. Children and young people are additionally adjusted for % not born in the United Kingdom and neighborhood English language proficiency. Wider barriers are additionally adjusted for neighborhood income, neighborhood English language proficiency, neighborhood % not born in the United Kingdom, and urban/rural. Geographical barriers are additionally adjusted for neighborhood income, neighborhood % not born in the United Kingdom, urban/rural, and air pollution. Crime is additionally adjusted for neighborhood income, neighborhood % not born in the United Kingdom, neighborhood English language proficiency, and urban/rural.

**FIGURE 3:**
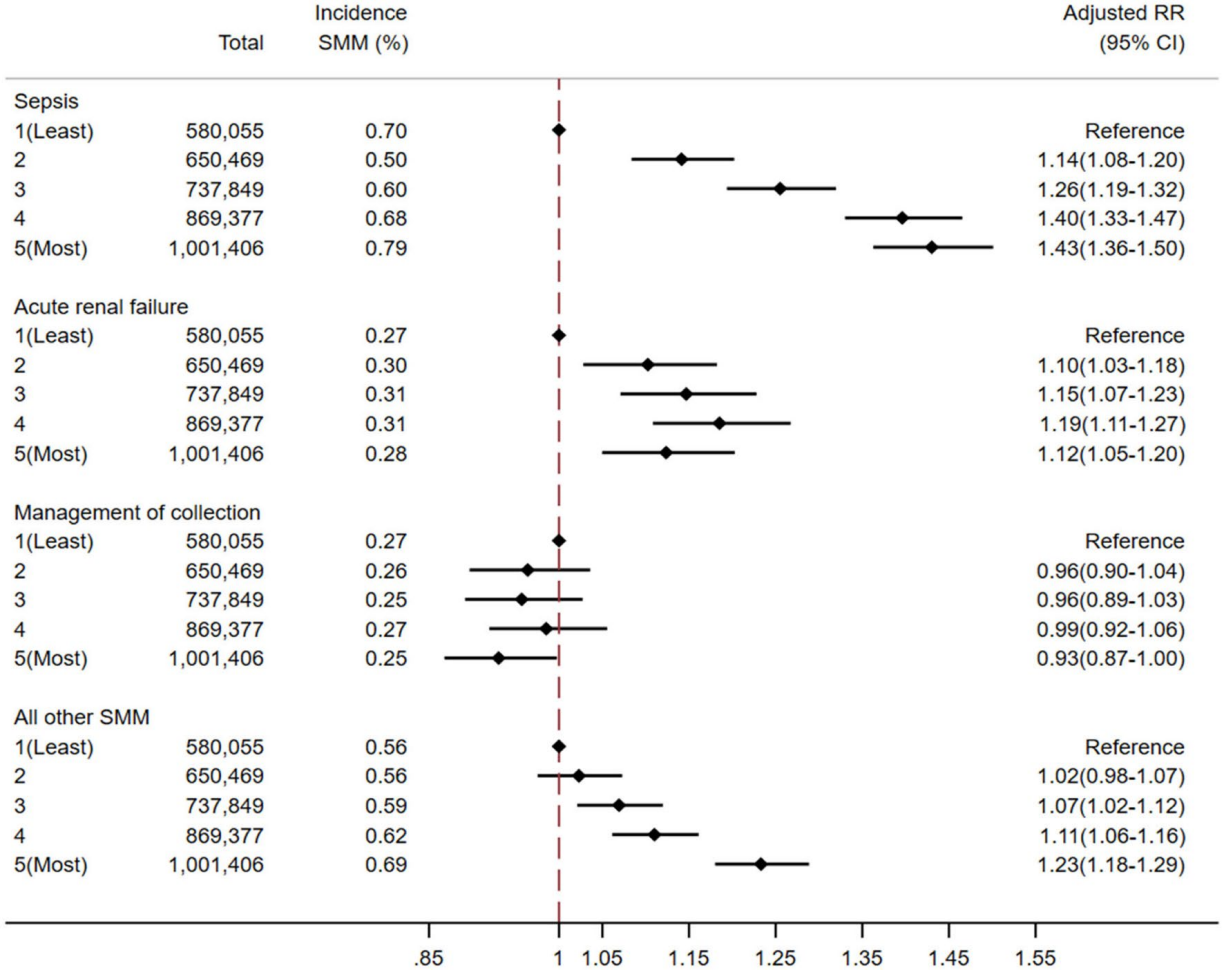
Adjusted risk ratio and 95% confidence interval (RR (95% CI)) of sepsis, acute renal failure, management of collection and “all other SMM” conditions in each of the composite IMD quintiles compared to the least deprived quintile. Adjusted for age, year, parity and ethnicity.

There was a dose–response relationship between composite IMD and SMM risk in Model 1 (Table S5, Figure 2), with an adjusted risk ratio of 1.21 (95% CI 1.17–1.24) for the most deprived IMD quintile compared to the least. Women in the most deprived areas faced higher risks of sepsis, acute renal failure, and “all other SMM conditions,” but not pelvic or intra‐abdominal collection management (Figure 3). A detailed breakdown of “all other SMM conditions” (Figure S5) showed a dose–response with increasing deprivation associated with higher risks of acute cardiac or embolic events, with embolic events having the strongest association (aRR 1.97, 95% CI 1.69–2.29) for women living in the most compared to the least deprived IMD quintile.

Univariable and multivariable multilevel model results for nine domains/subdomains are shown in Tables S6–S14. There was a dose–response relationship between the domains/subdomains, neighborhood income, employment, outdoor living environment, and adult skills and SMM, although the risk associated with the outdoor living environment was small (aRR: 1.06, 95% CI 1.03–1.10) (Figure 2). The subdomain, geographical barriers, was associated with an 8% lower risk of SMM (95% CI 0.89–0.95) for women living in the most deprived neighborhoods compared to the least. The subdomain, wider barriers, was associated with a 14% increased risk for SMM in the most deprived quintile (aRR 1.14, 95% CI 1.10–1.20). There was no evidence of an association between crime and SMM.

### 3.3 Effect modification by ethnicity

Ethnicity modified the association between both neighborhood education domains and SMM (adult skills (P_interaction_ = 0.048), and children and young people (P_interaction_ = 0.01), and SMM) but not for other domains/subdomains. Figures S6 and S7 show average adjusted predictive probabilities for each ethnic group by neighborhood education quintile. For White women, there was a dose–response relationship between lower adult skills and children's educational attainment and SMM risk with predicted probabilities of 1.65% (95% CI 1.62%–1.69%) and 1.59% (95% CI 1.56%–1.62%), respectively, in the most deprived quintiles. Predictive probabilities were higher for all minoritized groups than for White women across all educational attainment quintiles. Women of Bangladeshi origin living in areas with the highest neighborhood adult skills and second‐highest neighborhood children's educational attainment had the highest SMM risk, with predicted probabilities of 3.46% (95% CI 2.93%–4.00%) and 2.93% (95% CI 2.44%–3.42%), respectively.

### 3.4 Population attributable fractions (PAFs)

Socioeconomic deprivation measured using the composite IMD accounted for 11.4% (95% CI 9.5%–13.2%) of SMM. The domains, neighborhood income and employment, had the highest PAFs at 8.2% (95% CI 6.1%–10.3%) and 7.8% (95% CI 5.8%–9.8%), respectively (Table 1). About 20% (95% CI 17.7%–21.4%) of SMM could be attributed to socioeconomic deprivation (measured using the composite IMD) and ethnic inequality (Table 1). Among IMD domains and ethnic inequality combined, neighborhood income and employment again showed the highest PAFs at 16.5% (95% CI 14.4%–18.6%) and 15.9% (95% CI 13.9%–17.9%), respectively (Table 1).

**TABLE 1:** Univariable and multivariable population attributable fractions (PAFs) and their 95% confidence intervals (95% CI) of severe maternal morbidity (SMM) for the composite Index of Multiple Deprivation (IMD) and each domain/subdomain of IMD using both the least deprived 20% of women across all ethnicities and the least deprived 20% of White women as the baseline group.

	PAF (95% CI)									
	Income	Employment	Crime	Adult skills	Children and young people	Geographical barriers	Wider barriers	Indoor living environment	Outdoor living environment	Composite
Univariable analysis using the least deprived 20% of White women as the baseline group	17.2 (15.2‐19.1)	15.9 (14.0–17.8)	16.6 (14.7–18.5)	10.6 (8.6–12.5)	12.0 (10.0–14.0)	1.7 (−0.0–3.6)	17.7 (15.9–19.5)	15.5 (13.7–17.3)	15.8 (14.1–17.5)	17.8 (15.9–19.7)
Multivariable analysis using the least deprived 20% of White women as the baseline group	16.5 (14.4–18.6)	15.9 (13.9–17.9)	8.7 (5.6–10.5)	15.0 (13.1–16.9)	12.1 (9.9–14.2)	5.1 (3.1–7.0)	13.5 (11.0–15.9)	13.9 (12.0–15.7)	11.2 (9.0–13.2)	19.6 (17.7–21.4)
Univariable analysis using the least deprived 20% of all women as the baseline group	11.9 (10.0–13.8)	9.4 (7.5–11.2)	12.6 (10.7–14.4)	0.8 (−1.1–2.7)	3.9 (1.9–5.8)	−16.5 (−18.1 to −14.9)	15.2 (13.4–16.9)	7.4 (5.7–9.3)	13.3 (11.5–15.0)	13.2 (11.4–15.1)
Multivariable analysis using the least deprived 20% of all women as the baseline group	8.2 (6.1–10.3)	7.8 (5.8–9.8)	−1.1 (−3.7–1.4)	5.0 (3.2–6.8)	4.3 (2.3–6.2)	−4.5 (−6.1 to −2.9)	5.0 (2.4–7.5)	3.6 (1.6–5.4)	2.9 (1.0–5.1)	11.4 (9.5–13.2)

*Note*: The multivariable analysis uses Model 2 from each domain.

## 4 DISCUSSION

This study used routinely collected hospital admissions data of 4 040 106 maternities from 2013 to 2023 in England and showed an overall incidence of SMM of 17.7 per 1000 maternities. Income and employment had the strongest association with SMM among the individual IMD domains/subdomains. If all women faced the same risks as those living in the highest‐income neighborhoods or in areas with the highest level of employment, 8% of SMM could be reduced. The relationship between SMM and the education, skills and training domain varied across ethnic groups, where a dose–response association was observed only for White women. The proportion of SMM attributable to ethnic and socioeconomic disadvantage was approximately double that of the proportion attributable to disadvantage alone, highlighting the importance of considering both ethnic and socioeconomic inequalities when designing interventions to reduce SMM.

During the study period, the incidence rate of SMM increased from 8.7 (95% CI 8.5–9.0) per 1000 maternities in 2013 to 27.8 (95% CI 27.2–28.9) per 1000 maternities in 2022. A possible explanation for this increase could be that the population of women giving birth is becoming older, more obese, and with a greater number of medical complexities.^12^, ^13^ This could be due to a greater number of medical interventions, such as induction of labor or cesarean birth.^14^, ^15^ It was not possible to determine if this increase was due to a true increase in clinical cases, increased recognition, or a change in coding practices. Over time, the SMM risk gap between the most and least deprived quintiles appeared to widen, with the most significant increase in risk observed in the most deprived and second most deprived groups. This trend is also reflected in the most recent confidential enquiry into maternal deaths in England (2021–2023) where there was an increase (although not statistically significant one) in the disparities in maternal deaths in the most and least deprived neighborhoods compared to the 2019–2021 report.^1^

This study showed that sepsis, acute cardiac events, and embolism play a key role in the association between deprivation and SMM, with women living in the most deprived areas having a risk ratio of 1.43, 1.24, and 1.97 for each of the conditions, respectively, compared to women living in the least deprived areas. This offers a more nuanced understanding of the relationship between deprivation and SMM than has been described in previous studies, and is a key focus for policy intervention.^1^, ^2^ The association between living in the most deprived area and sepsis could be driven by factors such as differences in antenatal care uptake and continuity,^16^ delayed medical attention,^17^, ^18^ increased risk of pre‐existing conditions such as diabetes and hypertension^19^ and the increased labor induction rate^19^, ^20^ for women living in deprived areas, all increasing susceptibility to infections. Poor housing and overcrowded living conditions in deprived areas may also contribute to infection risks.^21^, ^22^ Women living in deprived communities were also more likely to be obese and to smoke, which are both associated with thromboembolism^23^ and cardiac events,^24^ which may explain the increased risk of these conditions.

This study highlighted the limitations of using a composite deprivation measure, such as the IMD, to study the association between socioeconomic disadvantage, and SMM. The strength of association varied by different aspects of neighborhood deprivation. Notably, neighborhood income and employment appeared to have the strongest association with SMM, with SMM risk increasing proportionally to decreasing relative neighborhood income and employment. Other studies have also found an association between neighborhood income and SMM, indicating that low income could play a crucial role in the relationship between deprivation and adverse maternal health outcomes.^4^ There was also a dose–response relationship between the outdoor living environment and the risk of SMM, although this effect was small. This may reflect that the environment has less of an immediate impact on SMM than material resources. The findings concerning housing issues and SMM, measured using the wider barriers subdomain, align with the results from a study in the United States (US)^25^ that reported a 73% increase in the risk of SMM for women living in a municipality with a higher rent to income ratio. However, in our study, an association between wider barriers and SMM was only seen in the most deprived quintile suggesting that housing problems may only affect SMM risk at the extreme end of the spectrum. However, the lack of observed associations in the other quintiles could also be due to power limitations in detecting smaller effects in less deprived groups.

Contrastingly, in comparison to the other domains/subdomains, areas with high geographical barriers to services were associated with a lower risk of SMM, with women living closest to services showing a higher risk. These findings may reflect that urban areas with better access to amenities and services also tend to experience higher poverty levels and social problems.^26^, ^27^ These factors may lead to a higher concentration of high‐risk pregnancies in these areas, which in turn increases the risk of SMM. In contrast, although women in rural areas may have less access to healthcare, they may encounter fewer social challenges, explaining the lower overall risk despite limited resources.^28^ An implication of the inverse relationship between the subdomain, geographical barriers, and SMM risk could be that it may dilute the impact of other dimensions of deprivation when combined into a single composite score, potentially masking important disparities.

In addition, the study demonstrated evidence for effect modification by ethnicity only for neighborhood education and SMM, but not for any of the other disadvantage domains, highlighting a further disadvantage of using a composite measure of deprivation. The dose–response relationship observed among women of White ethnicity for neighborhood education, skills, and training was not observed for women in any of the minoritized ethnic groups. This pattern was observed in both the children and young people subdomain, which reflects school attainment and access to higher education, and the adult skills subdomain, which includes neighborhood English language proficiency and adult qualifications. One possible explanation is that minoritized ethnic women may face additional barriers, such as discrimination and limited access to culturally appropriate care,^6^ regardless of their education, skills, or training. Furthermore, due to migration patterns, the level of socioeconomic disadvantage may be more likely to be misclassified for minoritized ethnic women than White women. This is especially true in the children and young people subdomain, which measures school attainment, where women from minoritized ethnic groups who migrated as adults may have had different educational experiences than younger generations born in the area. This finding has important implications for studies that use neighborhood education levels as a proxy for disadvantage in diverse communities.

The strengths and limitations of using a large‐population‐based administrative database and a composite outcome of SMM have been described elsewhere.^2^ To our knowledge, this study is the first to explore how various forms of deprivation are associated with SMM, which provides a more detailed understanding of the neighborhood factors most closely linked to SMM. Combining all Asian or all Black women into broader categories may oversimplify the socioeconomic, cultural, and biological differences within these groups.^29^ Categorizing ethnicity into ten subgroups allowed for a greater understanding of the intersection of socioeconomic disadvantage with ethnicity. Additionally, this is the first study to disaggregate different components of SMM, offering valuable information on which specific conditions should be targeted to reduce disparities in SMM.

However, a significant limitation of the study is the reliance on area‐level deprivation scores. While area‐level measures are useful for understanding contextual deprivation, a key issue is the risk of misclassification if they are used as proxies for individual‐level socioeconomic disadvantage, where women who are not socioeconomically disadvantaged may still live in deprived areas. Another limitation of area‐based measures is that they may conceal how socioeconomic disadvantage interacts with ethnicity and migration status. Migrants or individuals from ethnic minorities who do not experience socioeconomic disadvantage in the traditional sense based on income, education, or occupation may still reside in areas of high deprivation due to patterns of migration or settlement.^30^ The small numbers of certain ethnic groups living in the least deprived areas may have resulted in insufficient power to detect an association. This study was further limited as migration status was not available in the data to explore this in more detail. Another study limitation is the significant amount of missing data on gestational age at birth and birthweight, variables which were used to define the cohort of ≥20 weeks. There was also missing data on important confounders such as parity and the risk of false negatives of the outcome coded using ICD‐10 and OPCS‐4 codes, the impact of which is discussed in detail elsewhere.^8^ There was also missing data on ethnicity, which could introduce bias because ethnicity is associated with both deprivation and SMM. However, given that the proportion of missing data was small (<5%), any resulting impact on the findings is likely to be minimal. Finally, women with a gestational age at birth <20 weeks could not be included because the HES APC lacks a reliable denominator for this group; pregnancy losses before 20 weeks are not consistently captured in hospital records. As spontaneous or induced abortion may be associated with both the exposure and the outcome, this could introduce bias. Nonetheless, most SMM conditions occur after 20 weeks' gestation, and the EMMOI was specifically designed for this population, reducing the likelihood of major impact.

## 5 CONCLUSION

There appears to be a widening gap in the risk of SMM between women living in the least and most deprived areas in England, with sepsis, cardiac events, and embolism showing the strongest association with deprivation. However, the use of composite area‐level deprivation measures may obscure the varying impacts of different aspects of deprivation aspects how these intersect within different ethnic groups. Individual‐level data on socioeconomic disadvantage is crucial to better understand the pathways contributing to SMM risk and to develop targeted interventions to reduce the disparities in SMM demonstrated in this study.

## AUTHOR CONTRIBUTIONS

DGB conceptualized the project and undertook the literature review with support from RR and RG. DGB, RR, MK, and RG contributed to the study design. DGB and RR analyzed the data and all authors contributed to data interpretation and writing. All authors accept responsibility for the paper as published.

## FUNDING INFORMATION

RG and RR receive salary support from the Oxford NIHR Biomedical Research Centre. MK is an NIHR Senior Investigator (grant ref. NIHR303806). The views expressed in this publication are those of the author (s) and not necessarily those of the NHS, the NIHR, or the Department of Health and Social Care. The study sponsor and funder played no role in study design; in the collection, analysis and interpretation of data; in the writing of the report; and in the decision to submit the article for publication. All authors are independent of all funders.

## CONFLICT OF INTEREST STATEMENT

The authors declare no conflicts of interest.

## ETHICS STATEMENT

Under the assessment of the NHS Health Research Authority, using the HES APC data to conduct epidemiological and health service research at the University of Oxford does not need research ethics committee approval, as it is anonymized data.

## Supporting information


**Table S1.** ICD‐10 and OPCS‐4 for the modified English Maternal Morbidity Outcome Indicator (EMMOI).^4^
**Table S2.** ICD‐10 codes for covariates.
**Table S3.** Characteristics of the study population stratified by composite Index of Multiple Deprivation quintile *N* (%).
**Table S4.** Pearson correlation coefficients between the different domains/subdomains of the Index of Multiple Deprivation.
**Table S5.** Univariable and multivariable analysis showing risk ratios and their 95% confidence intervals (RR (95% CI)) between the composite Index of Multiple Deprivation (IMD) quintiles and severe maternal morbidity (SMM) compared to the least deprived IMD quintile.
**Table S6.** Univariable and multivariable analysis showing risk ratios and their 95% confidence intervals (RR (95% CI)) between income quintiles and severe maternal morbidity (SMM) compared to the least deprived income quintile.
**Table S7.** Univariable and multivariable analysis showing risk ratios and their 95% confidence intervals (RR (95% CI)) between employment quintiles and severe maternal morbidity (SMM) compared to the least deprived employment quintiles.
**Table S8.** Univariable and multivariable analysis showing risk ratios and their 95% confidence intervals (RR (95% CI)) between indoor living environment quintiles and severe maternal morbidity (SMM) compared to the least deprived indoor living environment quintile.
**Table S9.** Univariable and multivariable analysis showing risk ratios and their 95% confidence intervals (RR (95% CI)) between outdoor living environment quintiles and severe maternal morbidity (SMM) compared to the least deprived outdoor living environment quintile.
**Table S10.** Univariable and multivariable analysis showing risk ratios and their 95% confidence intervals (RR (95% CI)) between adult skills quintiles and severe maternal morbidity (SMM) compared to the least deprived adult skills quintile.
**Table S11.** Univariable and multivariable analysis showing risk ratios and their 95% confidence intervals (RR (95% CI)) between children and young people quintiles and severe maternal morbidity (SMM) compared to the least deprived children and young people quintile.
**Table S12.** Univariable and multivariable analysis showing risk ratios and their 95% confidence intervals (RR (95% CI)) between wider barriers quintiles and severe maternal morbidity (SMM) compared to the least deprived wider barriers quintile.
**Table S13.** Univariable and multivariable analysis showing risk ratios and their 95% confidence intervals (RR (95% CI)) between geographical barriers quintiles and severe maternal morbidity (SMM) compared to the least deprived geographical barriers quintile.
**Table S14.** Univariable and multivariable analysis showing risk ratios and their 95% confidence intervals (RR (95% CI)) between crime quintiles and severe maternal morbidity (SMM) compared to the least deprived crime quintile.
**Figure S1.** Flow chart showing the identification of the cohort.
**Figure S2.** The crude incidence rate (*N* per 1000 maternities) of severe maternal morbidity (SMM) over time by five ethnic groups.
**Figure S3.** The crude incidence rate (*N* per 1000 maternities) of severe maternal morbidity (SMM) over time by ten ethnic groups.
**Figure S4.** The proportion (%) of each severe maternal morbidity (SMM) diagnosis (ICD‐10) or procedure (OPCS‐4) in the final composite outcome.
**Figure S5.** Adjusted risk ratio and their 95% confidence intervals (RR [95% CI]) of conditions which make up the largest proportion of “all other SMM,” in each of the composite Index of Multiple Deprivation (IMD) quintiles compared to the least deprived quintile.
**Figure S6.** The average adjusted predictive margins and their 95% confidence intervals (95% CI) of severe maternal morbidity (SMM) for women living in each adult skills quintile stratified by ethnicity.
**Figure S7.** The average adjusted predictive margins and their 95% confidence intervals (95% CI) of severe maternal morbidity (SMM) for women living in each children and young people quintile stratified by ethnicity.

## Data Availability

Data may be obtained from a third party and are not publicly available. The data extract was derived from the English National Hospital Episode Statistics Admitted Patient Care (HES‐APC) database with linkage to national mortality civil registrations (https://digital.nhs.uk/data‐and‐information/data‐tools‐and‐services/dataservices/linked‐hes‐ons‐mortality‐data). Linked HES‐APC and mortality data are available upon application to NHS England (formerly NHS Digital).

## References

[1] Felker A , Knight M . MBRRACE‐UK update: key messages from the UK and Ireland confidential enquiries into maternal death and morbidity 2023. Obstet Gynaecol. 2024;26(1):27‐31.

[2] Geddes‐Barton D , Ramakrishnan R , Knight M , Goldacre R . Associations between neighbourhood deprivation, ethnicity and maternal health outcomes in England: a nationwide cohort study using routinely collected healthcare data. J Epidemiol Community Health. 2024;78:500‐507. 38834232 10.1136/jech-2024-222060 PMC11287519

[3] Geddes‐Barton DM , Ramakrishnan R , Knight M , Goldacre R . The Effect of Neighbourhood Deprivation and Ethnicity on Maternal Health Outcomes in England: A Nationwide Cohort Study Using Routinely Collected Healthcare Data. Available at SSRN 4561802. 10.1136/jech-2024-222060 PMC11287519 38834232

[4] Geddes‐Barton D , Baldelli S , Karthikappallil R , et al. Association between socioeconomic disadvantage and severe maternal morbidity and mortality in high‐income countries: a systematic review. J Epidemiol Community Health. 2025;79(3):207‐215. 39516003 10.1136/jech-2024-222407 PMC11874458

[5] Shavers VL . Measurement of socioeconomic status in health disparities research. J Natl Med Assoc. 2007;99(9):1013. 17913111 PMC2575866

[6] Vousden N , Bunch K , Kenyon S , Kurinczuk JJ , Knight M . Impact of maternal risk factors on ethnic disparities in maternal mortality: a national population‐based cohort study. Lancet Reg Health Eur. 2024;40:100893. 38585675 10.1016/j.lanepe.2024.100893 PMC10998184

[7] Herbert A , Wijlaars L , Zylbersztejn A , Cromwell D , Hardelid P . Data resource profile: hospital episode statistics admitted patient care (HES APC). Int J Epidemiol. 2017;46(4):1093. 28338941 10.1093/ije/dyx015 PMC5837677

[8] Geddes‐Barton D , Goldacre R , Knight M , Vousden N , Ramakrishnan R . Ethnic disparities in severe maternal morbidity and the contribution of deprivation: a population‐based causal analysis. BJOG. 2025;132:2131‐2137. 40511482 10.1111/1471-0528.18254 PMC12592783

[9] Benchimol EI , Smeeth L , Guttmann A , et al. The REporting of studies conducted using observational routinely‐collected health data (RECORD) statement. PLoS Med. 2015;12(10):e1001885. 26440803 10.1371/journal.pmed.1001885 PMC4595218

[10] Nair M , Kurinczuk JJ , Knight M . Establishing a national maternal morbidity outcome indicator in England: a population‐based study using routine hospital data. PLoS One. 2016;11(4):e0153370. 27054761 10.1371/journal.pone.0153370 PMC4824476

[11] D'Arcy RS . Investigating the Health and Care Needs of Pregnant Women with Multiple Long‐Term Conditions [PhD thesis (awaiting publication)]. 2024.

[12] Mills TA , Lavender T . Advanced maternal age. Obstet Gynaecol Reprod Med. 2011;21(4):107‐111.

[13] Heslehurst N , Rankin J , Wilkinson JR , Summerbell CD . A nationally representative study of maternal obesity in England, UK: trends in incidence and demographic inequalities in 619 323 births, 1989–2007. Int J Obes. 2010;34(3):420‐428. 10.1038/ijo.2009.250 20029373

[14] Bragg F , Cromwell DA , Edozien LC , et al. Variation in rates of caesarean section among English NHS trusts after accounting for maternal and clinical risk: cross sectional study. BMJ. 2010;341:c5065. 10.1136/bmj.c5065 PMC2950923 20926490

[15] Taylor B , Cross‐Sudworth F , Rimmer M , et al. Induction of labour care in the UK: a cross‐sectional survey of maternity units. PLoS One. 2024;19(2):e0297857. 38416750 10.1371/journal.pone.0297857 PMC10901341

[16] Acosta C , Bhattacharya S , Tuffnell D , Kurinczuk J , Knight M . Maternal sepsis: a Scottish population‐based case–control study. BJOG. 2012;119(4):474‐483. 22251396 10.1111/j.1471-0528.2011.03239.x PMC3328752

[17] Minejima E , Wong‐Beringer A . Impact of socioeconomic status and race on sepsis epidemiology and outcomes. J Appl Lab Med. 2021;6(1):194‐209. 33241269 10.1093/jalm/jfaa151

[18] Bladon S , Ashiru‐Oredope D , Cunningham N , et al. Rapid systematic review on risks and outcomes of sepsis: the influence of risk factors associated with health inequalities. Int J Equity Health. 2024;23(1):34. 38383380 10.1186/s12939-024-02114-6 PMC10882893

[19] Acosta CD , Harrison DA , Rowan K , Lucas DN , Kurinczuk JJ , Knight M . Maternal morbidity and mortality from severe sepsis: a national cohort study. BMJ Open. 2016;6(8):e012323. 10.1136/bmjopen-2016-012323 PMC5013336 27554107

[20] Carter S , Channon A , Berrington A . Socioeconomic risk factors for labour induction in the United Kingdom. BMC Pregnancy Childbirth. 2020;20:1‐13. 10.1186/s12884-020-2840-3 PMC7059288 32143597

[21] Lusk JB , Blass B , Mahoney H , et al. Neighborhood socioeconomic deprivation, healthcare access, and 30‐day mortality and readmission after sepsis or critical illness: findings from a nationwide study. Crit Care. 2023;27(1):287. 37454127 10.1186/s13054-023-04565-9 PMC10349422

[22] Hawker JI , Olowokure B , Sufi F , Weinberg J , Gill N , Wilson RC . Social deprivation and hospital admission for respiratory infection:: an ecological study. Respir Med. 2003;97(11):1219‐1224. 14635977 10.1016/s0954-6111(03)00252-x

[23] Anderson JFA , Spencer FA . Risk factors for venous thromboembolism. Circulation. 2003;107(23_suppl_1):I‐9‐I‐16. 10.1161/01.CIR.0000078469.07362.E6 12814980

[24] Mozaffarian D , Wilson PW , Kannel WB . Beyond established and novel risk factors: lifestyle risk factors for cardiovascular disease. Circulation. 2008;117(23):3031‐3038. 18541753 10.1161/CIRCULATIONAHA.107.738732

[25] Muchomba FM , Teitler J , Reichman NE . Association between housing affordability and severe maternal morbidity. JAMA Netw Open. 2022;5(11):e2243225. 36413368 10.1001/jamanetworkopen.2022.43225 PMC9682423

[26] Riva M , Curtis S , Gauvin L , Fagg J . Unravelling the extent of inequalities in health across urban and rural areas: evidence from a national sample in England. Soc Sci Med. 2009;68(4):654‐663. 19108940 10.1016/j.socscimed.2008.11.024 PMC5148618

[27] Lupton R , Fuller C . Mixed communities: a new approach to spatially concentrated poverty in England. Int J Urban Reg Res. 2009;33(4):1014‐1028.

[28] Smith I , Green E , Whittard D , Ritchie F . Re‐thinking the English indices of multiple deprivation: A review and exploration of alternative and complementary area‐based indicator systems. 2018.

[29] Villarroel N , Davidson E , Pereyra‐Zamora P , Krasnik A , Bhopal RS . Heterogeneity/granularity in ethnicity classifications project: the need for refining assessment of health status. Eur J Pub Health. 2019;29(2):260‐266. 30260371 10.1093/eurpub/cky191

[30] Shankley W , Hannemann T , Simpson L . The demography of ethnic minorities in Britain. Ethnicity, Race and Inequality in the UK. Policy Press; 2020:15‐34.

