# Supplementary material for: The association between different aspects of socioeconomic deprivation and severe maternal morbidity
Source: Acta Obstet Gynecol Scand. 2025 Dec 29;105(2):247–56. doi: 10.1111/aogs.70134 (PMC12856710; doi:10.1111/aogs.70134)
Supplement: Supplementary file 1 — Table S1. ICD‐10 and OPCS‐4 for the modified English Maternal Morbidity Outcome Indicator (EMMOI). 4 Table S2. ICD‐10 codes for covariates. Table S3. Characteristics of the study population stratified by composite Index of Multiple Deprivation quintile N (%). Table S4. Pearson correlation coefficients between the different domains/subdomains of the Index of Multiple Deprivation. Table S5. Univariable and multivariable analysis showing risk ratios and their 95% confidence intervals (RR (95% CI)) between the composite Index of Multiple Deprivation (IMD) quintiles and severe maternal morbidity (SMM) compared to the least deprived IMD quintile. Table S6. Univariable and multivariable analysis showing risk ratios and their 95% confidence intervals (RR (95% CI)) between income quintiles and severe maternal morbidity (SMM) compared to the least deprived income quintile. Table S7. Univariable and multivariable analysis showing risk ratios and their 95% confidence intervals (RR (95% CI)) between employment quintiles and severe maternal morbidity (SMM) compared to the least deprived employment quintiles. Table S8. Univariable and multivariable analysis showing risk ratios and their 95% confidence intervals (RR (95% CI)) between indoor living environment quintiles and severe maternal morbidity (SMM) compared to the least deprived indoor living environment quintile. Table S9. Univariable and multivariable analysis showing risk ratios and their 95% confidence intervals (RR (95% CI)) between outdoor living environment quintiles and severe maternal morbidity (SMM) compared to the least deprived outdoor living environment quintile. Table S10. Univariable and multivariable analysis showing risk ratios and their 95% confidence intervals (RR (95% CI)) between adult skills quintiles and severe maternal morbidity (SMM) compared to the least deprived adult skills quintile. Table S11. Univariable and multivariable analysis showing risk ratios and their 95% confidence intervals (RR (95% CI [file AOGS-105-247-s001.docx]

**Supplementary Methods**

**Index of Multiple Deprivation**

The Index of Multiple Deprivation (IMD) is widely used in England to measure multidimensional neighbourhood socioeconomic deprivation (1, 2). It is a composite measure comprising seven domains (income, employment, education, health, crime, barriers to housing and services, and living environment). Each domain is assigned a weighted value relative to its importance in overall deprivation. This study selected deprivation at the smallest neighbourhood measure of lower layer super output areas (LSOAs) which are neighbourhoods of approximately 1,500 people. The overall deprivation level in an LSOA is measured by the extent to which people within the LSOA experience different domains of deprivation, which are measured by separate indices. The areas (LSOAs) are then ranked nationally, with 1 being the least deprived and 32,844 being the most. Higher scores on any deprivation domain indicate more deprived areas. Exposures were defined based on the 2019 and 2015 IMD releases. Births before January 1, 2015, used the 2015 IMD, while births on or after that date used the 2019 IMD. Domains/subdomains included income, employment, adult skills, children and young people, geographical barriers, wider barriers, indoor living environment, outdoor living environment, and crime (**Table S1)**.

**Covariates**

Age was modelled as a categorical variable in 5-year groups due to evidence for departure from linearity (p<0.001). Ethnicity followed the Office for National Statistics (ONS) categorisation (3), collapsed into ten groups: White, Black or Black British African, Black or Black British Caribbean, Asian or Asian British Indian, Asian or Asian British Pakistani, Asian or Asian British Bangladeshi, Mixed, Other, Other Black, and Other Asian. Parity was categorised as nulliparous (yes/no). Data on pre-existing medical conditions, mental health, and smoking/ substance misuse were obtained from ICD-10 codes (**Table S2**). The year refers to the baby's birth year. Neighbourhood country of birth, adult qualifications, and English language proficiency were derived from ONS 2011 census data at LSOA level. Proportions were calculated, and LSOAs were ranked accordingly. The ONS Rural Urban Classification distinguished rural and urban areas using 2011 LSOAs.

**Table S1.** ICD-10 and OPCS-4 for the modified English Maternal Morbidity Outcome Indicator (EMMOI)(4)

| **Morbid event category (ICD-10 codes)** | **Codes** |
| --- | --- |
| Acute abdomen | N733 Female acute pelvic peritonitis  N735 Female pelvic peritonitis, unspecified  K650 Acute peritonitis  K659 Peritonitis, unspecified  K35 Acute appendicitis  K352 Acute appendicitis with generalized peritonitis  K353 Acute appendicitis with localized peritonitis  K358 Acute appendicitis, other and unspecified  K37 Unspecified appendicitis  K562 Volvulus  K565 Intestinal adhesions (bands) with obstruction  K566 Other and unspecified intestinal obstruction  K593 Megacolon, not elsewhere classified |
| Acute renal failure | O904 Postpartum acute renal failure  N17 Acute renal failure  N170 Acute renal failure with tubular necrosis  N171 Acute renal failure with acute cortical necrosis  N172 Acute renal failure with medullary necrosis  N178 Other acute renal failure  N179 Acute renal failure, unspecified  N19 Unspecified kidney failure  N990 Postprocedural renal failure  I120 Hypertensive renal disease with renal failure  I131 Hypertensive heart and renal disease with renal failure |
| Acute psychosis | F23 Acute and transient psychotic disorders  F230 Acute polymorphic psychotic disorder without symptoms of schizophrenia  F231 Acute polymorphic psychotic disorder with symptoms of schizophrenia  F232 Acute schizophrenia-like psychotic disorder  F238 Other acute and transient psychotic disorders  F239 Acute and transient psychotic disorder, unspecified  F531 Severe mental and behavioural disorders associated with the puerperium, not elsewhere classified |
| Acute cardiac event (cardiac infarction, cardiac failure, cardiomyopathy and cardiac arrest) | O903 Cardiomyopathy in the puerperium  I21 Acute myocardial infarction  I210 Acute transmural myocardial infarction of anterior wall  I211 Acute transmural myocardial infarction of inferior wall  I212 Acute transmural myocardial infarction of other sites  I213 Acute transmural myocardial infarction of unspecified site  I214 Acute subendocardial myocardial infarction  I219 Acute myocardial infarction, unspecified  I42 Cardiomyopathy  I420 Dilated cardiomyopathy  I421 Obstructive hypertrophic cardiomyopathy  I422 Other hypertrophic cardiomyopathy  I423 Endomyocardial (eosinophilic) disease  I424 Endocardial fibroelastosis  I425 Other restrictive cardiomyopathy  I426 Alcoholic cardiomyopathy  I427 Cardiomyopathy due to drugs and other external agents  I428 Other cardiomyopathies  I429 Cardiomyopathy, unspecified  I43* Cardiomyopathy in diseases classified elsewhere  I430* Cardiomyopathy in infectious and parasitic diseases classified elsewhere  I431* Cardiomyopathy in metabolic diseases  I432* Cardiomyopathy in nutritional diseases  I438* Cardiomyopathy in other diseases classified elsewhere  I46 Cardiac arrest  I460 Cardiac arrest with successful resuscitation  I461 Sudden cardiac death, so described  I469 Cardiac arrest, unspecified  I50 Heart failure  I500 Congestive heart failure  I501 Left ventricular failure  I509 Heart failure, unspecified  I110 Hypertensive heart disease with (congestive) heart failure  I119 Hypertensive heart disease without (congestive) heart failure  I130 Hypertensive heart and renal disease with (congestive) heart failure  I132 Hypertensive heart and renal disease with both (congestive) heart failure and renal failure |
| Acute respiratory compromise | J81 Pulmonary oedema  J80 Adult respiratory distress syndrome |
| Cerebral oedema or coma | G936 Cerebral oedema  R402 Coma, unspecified |
| Disseminated intravascular coagulopathy | O450 Premature separation of placenta with coagulation defect (including placental abruption with excessive haemorrhage associated with afibrinoginaemia, disseminated intravascular coagulation, hyperfibrinolysis, hyperfibrinogenaemia)  O460 Antepartum haemorrhage with coagulation defect (antepartum haemorrhage (excessive) associated with afibrinoginaemia, disseminated intravascular coagulation, hyperfibrinolysis, hyperfibrinogenaemia)  O670 Intrapartum haemorrhage with coagulation defect (intrapartum haemorrhage (excessive) associated with afibrinoginaemia, disseminated intravascular coagulation, hyperfibrinolysis, hyperfibrinogenaemia)  D65 Disseminated intravascular coagulation (defibrination syndrome) |
| Cerebrovascular accident | I60 Subarachnoid haemorrhage  I600 Subarachnoid haemorrhage from carotid siphon and bifurcation  I601 Subarachnoid haemorrhage from middle cerebral artery  I602 Subarachnoid haemorrhage from anterior communicating artery  I603 Subarachnoid haemorrhage from posterior communicating artery  I604 Subarachnoid haemorrhage from basilar artery  I605 Subarachnoid haemorrhage from vertebral artery  I606 Subarachnoid haemorrhage from other intracranial arteries  I607 Subarachnoid haemorrhage from intracranial artery, unspecified  I608 Other subarachnoid haemorrhage  I609 Subarachnoid haemorrhage, unspecified  I61 Intracerebral haemorrhage  I610 Intracerebral haemorrhage in hemisphere, subcortical  I611 Intracerebral haemorrhage in hemisphere, cortical  I612 Intracerebral haemorrhage in hemisphere, unspecified  I613 Intracerebral haemorrhage in brain stem  I614 Intracerebral haemorrhage in cerebellum  I615 Intracerebral haemorrhage, intraventricular  I616 Intracerebral haemorrhage, multiple localized  I618 Other intracerebral haemorrhage  I619 Intracerebral haemorrhage, unspecified  I62 Other nontraumatic intracranial haemorrhage  I620 Subdural haemorrhage (acute)(nontraumatic)  I621 Nontraumatic extradural haemorrhage  I629 Intracranial haemorrhage (nontraumatic), unspecified  I63 Cerebral infarction  I630 Cerebral infarction due to thrombosis of precerebral arteries  I631 Cerebral infarction due to embolism of precerebral arteries  I632 Cerebral infarction due to unspecified occlusion or stenosis of precerebral arteries  I633 Cerebral infarction due to thrombosis of cerebral arteries  I634 Cerebral infarction due to embolism of cerebral arteries  I635 Cerebral infarction due to unspecified occlusion or stenosis of cerebral arteries  I638 Other cerebral infarction  I639 Cerebral infarction, unspecified  I64 Stroke, not specified as haemorrhage or infarction |
| Major complications of anaesthesia | O740 Aspiration pneumonitis due to anaesthesia during labour and birth  O741 Other pulmonary complications of anaesthesia during labour and birth  O742 Cardiac complications of anaesthesia during labour and birth  O743 Central nervous system complications of anaesthesia during labour and birth  O749 Complication of anaesthesia during labour and birth, unspecified  O890 Pulmonary complications of anaesthesia during the puerperium  O891 Cardiac complications of anaesthesia during the puerperium  O892 Central nervous system complications of anaesthesia during the puerperium  O290 Pulmonary complications of anaesthesia during pregnancy  O291 Cardiac complications of anaesthesia during pregnancy  O292 Central nervous system complications of anaesthesia during pregnancy  O296 Failed or difficult intubation during pregnancy  O293 Toxic reaction to local anaesthesia during pregnancy |
| Embolic event (pulmonary embolism, amniotic fluid embolism, septic embolism and air embolism) | O88 Obstetric embolism  O880 Obstetric air embolism  O881 Amniotic fluid embolism  O882 Obstetric blood-clot embolism  O883 Obstetric pyaemic and septic embolism  O888 Other obstetric embolism  I26 Pulmonary embolism  I260 Pulmonary embolism with mention of acute cor pulmonale  I269 Pulmonary embolism without mention of acute cor pulmonale |
| Shock | R570 Cardiogenic shock  R571 Hypovolaemic shock  R572 Septic shock  R578 Other shock  R579 Shock, unspecified  O751 Shock during or following labour and birth  T805 Anaphylactic shock due to serum  T886 Anaphylactic shock due to adverse effect of correct drug or medicament properly administered  T782 Anaphylactic shock, unspecified  T780 Anaphylactic shock due to adverse food reaction  A483 Toxic shock syndrome |
| Sickle cell anaemia with crisis | D570 Sickle-cell anaemia with crisis |
| Status asthmaticus | J46 Status asthmaticus |
| Status epilepticus | G41 Status epilepticus  G410 Grand mal status epilepticus  G411 Petit mal status epilepticus  G412 Complex partial status epilepticus  G418 Other status epilepticus  G419 Status epilepticus, unspecified |
| Uterine rupture | O710 Rupture of uterus before onset of labour  O711 Rupture of uterus during labour |
| Eclampsia | O15 Eclampsia  O150 Eclampsia in pregnancy  O151 Eclampsia in labour  O152 Eclampsia in the puerperium  O159 Eclampsia, unspecified as to time period |
| Sepsis | O85 Puerperal sepsis  A40 Streptococcal sepsis  A400 Sepsis due to streptococcus, group A  A401 Sepsis due to streptococcus, group B  A402 Sepsis due to streptococcus, group D  A403 Sepsis due to Streptococcus pneumoniae  A408 Other streptococcal sepsis  A409 Streptococcal sepsis, unspecified  A41 Other sepsis  A410 Sepsis due to Staphylococcus aureus  A411 Sepsis due to other specified staphylococcus  A412 Sepsis due to unspecified staphylococcus  A413 Sepsis due to Haemophilus influenzae  A414 Sepsis due to anaerobes  A415 Sepsis due to other Gram-negative organisms  A418 Other specified sepsis  A419 Sepsis, unspecified (including septicaemia)  A327 Listerial sepsis |
| Cerebral venous thrombosis | O873 Cerebral venous thrombosis in the puerperium  I636 Cerebral infarction due to cerebral venous thrombosis, nonpyogenic  I676 Nonpyogenic thrombosis of intracranial venous system (including non-pyogenic thrombosis of cerebral vein and intracranial venous sinus) |
| Acute pancreatitis | K85 Acute pancreatitis  K850 Idiopathic acute pancreatitis  K851 Biliary acute pancreatitis  K852 Alcohol-induced acute pancreatitis  K853 Drug-induced acute pancreatitis  K858 Other acute pancreatitis  K859 Acute pancreatitis, unspecified  K863 Pseudocyst of pancreas |
| Rupture of aortic aneurysm or dissection of aorta | I710 Dissection of aorta (any part)  I711 Thoracic aortic aneurysm, ruptured  I713 Abdominal aortic aneurysm, ruptured  I715 Thoracoabdominal aortic aneurysm, ruptured  I718 Aortic aneurysm of unspecified site, ruptured  I722 Aneurysm and dissection of renal artery  I723 Aneurysm and dissection of iliac artery  I712 Thoracic aortic aneurysm, without mention of rupture  I714 Abdominal aortic aneurysm, without mention of rupture  I716 Thoracoabdominal aortic aneurysm, without mention of rupture  I719 Aortic aneurysm of unspecified site, without mention of rupture |
| Diabetic ketoacidosis | E100 Diabetes mellitus with coma (including hyperglycaemic coma NOS, diabetic coma with or without ketoacidosis, diabetic hyperosmolar coma, diabetic hypoglycaemic coma)  E101 Diabetes mellitus with ketoacidosis |
| **Morbid event category (OPCS-4 codes)** | **Codes** |
| Respiratory support | E851 Invasive ventilation (Includes endotracheal intermittent positive pressure ventilation) E852 Non-invasive ventilation NEC (Includes continuous positive airway pressure, intermittent positive pressure ventilation NEC, Negative pressure ventilation, Bilevel positive airway pressure, High flow continuous positive airway pressure)  E421 Permanent tracheostomy E422 Cricothyroidostomy E423 Temporary tracheostomy (includes tracheostomy NEC, Traccheostomy, Placement of tracheostomy tube) E428 Other specified (Exteriorisation of trachea) E429 Unspecified (Exteriorisation of trachea)  E856: Continuous positive airway pressure  E858: Other specified ventilation support  E859: Unspecified ventilation support  E899: Unspecified other respiratory support  E898: Other specified other respiratory support  Y733: Ventilatory support  Y731: Cardiopulmonary bypass  Y732: Extracorporeal circulation NEC |
| Surgical evacuation of the uterus following birth | R281 Curettage of delivered uterus  R288 Other specified (Instrumental removal of products of conception from delivered uterus) R289 Unspecified (Instrumental removal of products of conception from delivered uterus)  Q10 Curettage of uterus  Q101 Dilation of cervix uteri and curettage of products of conception from uterus  Q102 Curettage of products of conception from uterus NEC  Q103 Dilation of cervix uteri and curettage of uterus NEC  Q108 Other specified  Q109 Unspecified  Q11 Other evacuation of contents of uterus  Q111 Vacuum aspiration of products of conception from uterus NEC  Q115 Vacuum aspiration of products of conception from uterus using rigid cannula Q116 Vacuum aspiration of products of conception from uterus using flexible cannula  Q113 Evacuation of products of conception from uterus NEC |
| Dialysis | X40 Compensation for renal failure  X401 Renal dialysis  X402 Peritoneal dialysis NEC  X403 Haemodialysis NEC  X404 Haemofiltration X405 Automated peritoneal dialysis X406 Continuous ambulatory peritoneal dialysis X407 Haemoperfusion X408 Other specified X409 Unspecified X411 Insertion of ambulatory peritoneal dialysis catheter  X421 Insertion of temporary peritoneal dialysis catheter |
| Management of intra-abdominal or pelvic collection | T341 Open drainage of subphrenic abscess T342 Open drainage of pelvic abscess T343 Open drainage of abdominal abscess NEC  T451 Image controlled percutaneous drainage of subphrenic abscess T452 Image controlled percutaneous drainage of pelvic abscess T453 Image controlled percutaneous drainage of abdominal abscess NEC T454 Image controlled percutaneous drainage of lesion of abdominal cavity NEC  Y22 Drainage of organ NOC  Y221 Aspiration of haematoma of organ NOC  T348 Other specified (open drainage of peritoneum) T349 Unspecified (open drainage of peritoneum)  T468: Other specified other drainage of peritoneal cavity  T469: Unspecified other drainage of peritoneal cavity  T963: Debridement of soft tissue NEC  Y055: Debridement of organ NOC  Y228: Other specified drainage of organ NOC  Y229: Unspecified drainage of organ NOC  Y251: Suture of laceration of organ NOC  Y252: Resuture of organ NOC  Y321: Re-exploration of organ and surgical arrest of postoperative bleeding NOC  Y322: Re-exploration of organ and other repair of organ NOC  Y323: Re-exploration of organ and packing of organ NOC  Y328: Other specified re-exploration of organ NOC  Y329: Unspecified re-exploration of organ NOC  T301: Reopening of abdomen and re-exploration of intra-abdominal operation site and surgical arrest of postoperative bleeding  T302: Reopening of abdomen and re-exploration of intra-abdominal operation site NEC  T303: Reopening of abdomen NEC  H031: Drainage of abscess of appendix  H581: Drainage of ischiorectal abscess  H583: Drainage of perirectal abscess |
| Management of vulval or vaginal haematoma | P093 Evacuation of haematoma from vulva  P271 Evacuation of haematoma from vagina |
| Hysterectomy | Q074 Total abdominal hysterectomy NEC  Q075 Subtotal abdominal hysterectomy  R251 Caesarean hysterectomy |
| Interventional and surgical procedures to arrest major haemorrhage following birth | L702 Open embolisation of artery NEC  L703 Ligation of artery NEC  L713 Percutaneous transluminal embolisation of artery  L933 Ligation of vein NEC  L941 Percutaneous transluminal embolisation of vein  L995: Percutaneous transluminal occlusion of vein NEC  L531: Repair of iliac artery NEC  L538: Other specified other open operations on iliac artery  L539: Unspecified other open operations on iliac artery  L543: Arteriography of iliac artery  L663: Percutaneous transluminal occlusion of artery  L721: Arteriography NEC  L937: Repair of vein NEC  L974: Operations on artery NEC  L975: Operations on vein NEC  L974: Operations on artery NEC  L975: Operations on vein NEC  Y781: Arteriotomy approach to organ using image guidance with fluoroscopy  Y782: Arteriotomy approach to organ using image guidance with computed tomography  Y793: Transluminal approach to organ through femoral artery  Y788: Other specified arteriotomy approach to organ under image control  Y789: Unspecified arteriotomy approach to organ under image control  Y798: Other specified approach to organ through artery  Y799: Unspecified approach to organ through artery |
| Management of caesarean wound dehiscence | T283: Resuture of previous incision of anterior abdominal wall  T288: Other specified other repair of anterior abdominal wall  T289: Unspecified other repair of anterior abdominal wall |
| Repair of damage to bladder or urinary tract | M373: Repair of rupture of bladder  M378: Other specified other repair of bladder  M379: Unspecified other repair of bladder  M736: Urethroplasty NEC  M737: Repair of rupture of urethra NEC  M738: Other specified repair of urethra  M136: Percutaneous insertion of nephrostomy tube  M182: Excision of segment of ureter  M188: Other specified excision of ureter  M189: Unspecified excision of ureter  M191: Construction of ileal conduit  M192: Creation of urinary diversion to intestine NEC  M194: Cutaneous ureterostomy NEC  M201: Bilateral replantation of ureter  M202: Unilateral replantation of ureter  M203: Replantation of ureter after urinary diversion  M208: Other specified replantation of ureter  M209: Unspecified replantation of ureter  M211: Direct anastomosis of ureter to bladder  M212: Anastomosis of ureter to bladder using flap of bladder  M213: Ileal replacement of ureter  M214: Colonic replacement of ureter  M216: Ureteroureterostomy  M221: Suture of ureter  M222: Removal of ligature from ureter  M228: Other specified repair of ureter  M229: Unspecified repair of ureter  M274: Ureteroscopic insertion of ureteric stent  M277: Ureteroscopic dilation of ureter  M278: Other specified therapeutic ureteroscopic operations on ureter  M279: Unspecified therapeutic ureteroscopic operations on ureter  M294: Endoscopic dilation of ureter  M331: Percutaneous insertion of metallic stent into ureter  M332: Percutaneous insertion of plastic stent into ureter  M338: Other specified percutaneous ureteric stent procedures  M339: Unspecified percutaneous ureteric stent procedures  M734: Reconstruction of urethra  M738: Other specified repair of urethra  M739: Unspecified repair of urethra |
| Repair of damage to the intestine and management of intestinal obstruction | G581: Total jejunectomy and anastomosis of stomach to ileum  G582: Total jejunectomy and anastomosis of duodenum to ileum  G583: Total jejunectomy and anastomosis of duodenum to colon  G584: Partial jejunectomy and anastomosis of jejunum to ileum  G585: Partial jejunectomy and anastomosis of duodenum to colon  G588: Other specified excision of jejunum  G589: Unspecified excision of jejunum  G691: Ileectomy and anastomosis of stomach to ileum  G692: Ileectomy and anastomosis of duodenum to ileum  G693: Ileectomy and anastomosis of ileum to ileum  G694: Ileectomy and anastomosis of ileum to colon  G698: Other specified excision of ileum  G699: Unspecified excision of ileum  G784: Closure of perforation of ileum  G785: Exclusion of segment of ileum  G786: Open intubation of ileum  H061: Extended right hemicolectomy and end to end anastomosis  H062: Extended right hemicolectomy and anastomosis of ileum to colon  H069: Unspecified extended excision of right hemicolon  H071: Right hemicolectomy and end to end anastomosis of ileum to colon  H072: Right hemicolectomy and side to side anastomosis of ileum to transverse colon  H073: Right hemicolectomy and anastomosis NEC  H074: Right hemicolectomy and ileostomy HFQ  H075: Right hemicolectomy and end to side anastomosis  H078: Other specified other excision of right hemicolon  H079: Unspecified other excision of right hemicolon  H081: Transverse colectomy and end to end anastomosis  H082: Transverse colectomy and anastomosis of ileum to colon  H083: Transverse colectomy and anastomosis NEC  H084: Transverse colectomy and ileostomy HFQ  H085: Transverse colectomy and exteriorisation of bowel NEC  H086: Transverse colectomy and end to side anastomosis  H088: Other specified excision of transverse colon  H089: Unspecified excision of transverse colon  H091: Left hemicolectomy and end to end anastomosis of colon to rectum  H092: Left hemicolectomy and end to end anastomosis of colon to colon  H112: Colectomy and side to side anastomosis of ileum to colon NEC  H113: Colectomy and anastomosis NEC  H114: Colectomy and ileostomy NEC  H115: Colectomy and exteriorisation of bowel NEC  H116: Colectomy and end to side anastomosis NEC  H118: Other specified other excision of colon  H119: Unspecified other excision of colon  H291: Subtotal excision of colon and rectum and creation of colonic pouch and anastomosis of colon to anus  H292: Subtotal excision of colon and rectum and creation of colonic pouch NEC  H293: Subtotal excision of colon and creation of colonic pouch and anastomosis of colon to rectum  H294: Subtotal excision of colon and creation of colonic pouch NEC  H295: Subtotal excision of colon and anastomosis of colon to ileum  H298: Other specified subtotal excision of colon  H299: Unspecified subtotal excision of colon  H331: Abdominoperineal excision of rectum and end colostomy  H332: Proctectomy and anastomosis of colon to anus  H333: Anterior resection of rectum and anastomosis of colon to rectum using staples  H334: Anterior resection of rectum and anastomosis NEC  H335: Rectosigmoidectomy and closure of rectal stump and exteriorisation of bowel  H336: Anterior resection of rectum and exteriorisation of bowel  H337: Perineal resection of rectum HFQ  H338: Other specified excision of rectum  H339: Unspecified excision of rectum  T374: Repair of mesentery of small intestine  T384: Repair of mesentery of colon  H172: Open reduction of volvulus of caecum  H173: Open reduction of volvulus of sigmoid colon  H174: Open reduction of volvulus of colon NEC  H175: Open relief of strangulation of colon  H176: Open relief of obstruction of colon NEC  H158: Other specified other exteriorisation of colon  H159: Unspecified other exteriorisation of colon  H151: Loop colostomy  H152: End colostomy  H131: Bypass of colon by anastomosis of ileum to colon  H132: Bypass of colon by anastomosis of caecum to sigmoid colon  H133: Bypass of colon by anastomosis of transverse colon to sigmoid colon  H134: Bypass of colon by anastomosis of transverse colon to rectum  H135: Bypass of colon by anastomosis of colon to rectum NEC  H138: Other specified bypass of colon  H139: Unspecified bypass of colon  H101: Sigmoid colectomy and end to end anastomosis of ileum to rectum  H102: Sigmoid colectomy and anastomosis of colon to rectum  H103: Sigmoid colectomy and anastomosis NEC  H104: Sigmoid colectomy and ileostomy HFQ  H105: Sigmoid colectomy and exteriorisation of bowel NEC  H106: Sigmoid colectomy and end to side anastomosis  H108: Other specified excision of sigmoid colon  H109: Unspecified excision of sigmoid colon  H051: Total colectomy and anastomosis of ileum to rectum  H052: Total colectomy and ileostomy and creation of rectal fistula HFQ  H053: Total colectomy and ileostomy NEC  H058: Other specified total excision of colon  H059: Unspecified total excision of colon  G633: Closure of perforation of jejunum  G721: Anastomosis of ileum to caecum  G722: Anastomosis of ileum to transverse colon  G723: Anastomosis of ileum to colon NEC  G724: Anastomosis of ileum to rectum  G725: Anastomosis of ileum to anus and creation of pouch HFQ  G741: Creation of continent ileostomy  G742: Creation of temporary ileostomy  G743: Creation of defunctioning ileostomy  G762: Open relief of strangulation of ileum  G763: Open relief of obstruction of ileum NEC  T305: Packing of abdominal cavity |
| Management of acute coronary syndrome | K634: Coronary arteriography using two catheters  K635: Coronary arteriography using single catheter  K636: Coronary arteriography NEC  K638: Other specified contrast radiology of heart  K639: Unspecified contrast radiology of heart  K651: Catheterisation of combination of right and left side of heart NEC  K652: Catheterisation of right side of heart NEC  K653: Catheterisation of left side of heart NEC  K654: Catheterisation of left side of heart via atrial transeptal puncture  K658: Other specified catheterisation of heart  K659: Unspecified catheterisation of heart  K751: Percutaneous transluminal balloon angioplasty and insertion of 1-2 drug-eluting stents into coronary artery  K752: Percutaneous transluminal balloon angioplasty and insertion of 3 or more drug-eluting stents into coronary artery  K753: Percutaneous transluminal balloon angioplasty and insertion of 1-2 stents into coronary artery  K754: Percutaneous transluminal balloon angioplasty and insertion of 3 or more stents into coronary artery NEC  K758: Other specified percutaneous transluminal balloon angioplasty and insertion of stent into coronary artery  K759: Unspecified percutaneous transluminal balloon angioplasty and insertion of stent into coronary artery  L761: Endovascular placement of one metallic stent  L762: Endovascular placement of one plastic stent  L763: Endovascular placement of two metallic stents  L764: Endovascular placement of two plastic stents  L765: Endovascular placement of three or more metallic stents  L766: Endovascular placement of three or more plastic stents  L767: Endovascular placement of metallic stent with mechanical embolic protection  L768: Other specified endovascular placement of stent  L769: Unspecified endovascular placement of stent  L891: Endovascular placement of two drug-eluting stents  L892: Endovascular placement of two coated stents  L893: Endovascular placement of three or more drug-eluting stents  L894: Endovascular placement of three or more coated stents  L895: Endovascular placement of one drug-eluting stent  L896: Endovascular placement of one coated stent  L898: Other specified other endovascular placement of stent  L899: Unspecified other endovascular placement of stent |
| Interventional and surgical procedures to manage thromboembolism | L791: Insertion of filter into vena cava  L124: Open embolectomy of pulmonary artery  L131: Percutaneous transluminal embolectomy of pulmonary artery  L961: Percutaneous mechanical thromboembolectomy  L962: Percutaneous aspiration thromboembolectomy  L968: Other specified percutaneous removal of thrombus from vein  L969: Unspecified percutaneous removal of thrombus from vein  L993: Percutaneous transluminal venous thrombolysis with reconstruction  L994: Percutaneous transluminal venous thrombolysis NEC |
| Interventional and surgical procedures to manage major haemorrhage originating from the spleen | J691: Total excision of spleen and replantation of fragments of spleen  J692: Total splenectomy  J698: Other specified total excision of spleen  J699: Unspecified total excision of spleen  J701: Partial splenectomy  J722: Embolisation of spleen  J724: Repair of spleen  J725: Banding of spleen |
| Interventional procedures to treat haemorrhagic or ischaemic stroke | L331: Excision of aneurysm of cerebral artery  L332: Clipping of aneurysm of cerebral artery  L333: Ligation of aneurysm of cerebral artery NEC  L334: Obliteration of aneurysm of cerebral artery NEC  L338: Other specified operations on aneurysm of cerebral artery  L339: Unspecified operations on aneurysm of cerebral artery  L343: Open embolectomy of cerebral artery  L344: Open embolisation of cerebral artery  L351: Percutaneous transluminal embolisation of cerebral artery  L352: Arteriography of cerebral artery  L353: Percutaneous transluminal insertion of stent into cerebral artery  L354: Percutaneous transluminal embolectomy of cerebral artery  L358: Other specified transluminal operations on cerebral artery  L359: Unspecified transluminal operations on cerebral artery  L961: Percutaneous mechanical thromboembolectomy  L962: Percutaneous aspiration thromboembolectomy  O011: Percutaneous transluminal coil embolisation of small aneurysm of artery  O012: Percutaneous transluminal coil embolisation of medium aneurysm of artery  O013: Percutaneous transluminal coil embolisation of large aneurysm of artery  O014: Percutaneous transluminal coil embolisation of giant aneurysm of artery  O018: Other specified transluminal coil embolisation of aneurysm of artery  O019: Unspecified transluminal coil embolisation of aneurysm of artery  O021: Percutaneous transluminal balloon assisted coil embolisation of three or more aneurysms of artery  O022: Percutaneous transluminal balloon assisted coil embolisation of two aneurysms of artery  O023: Percutaneous transluminal balloon assisted coil embolisation of single aneurysm of artery  O028: Other specified transluminal balloon assisted coil embolisation of aneurysm of artery  O029: Unspecified transluminal balloon assisted coil embolisation of aneurysm of artery  O031: Percutaneous transluminal stent assisted coil embolisation of three or more aneurysms of artery  O032: Percutaneous transluminal stent assisted coil embolisation of two aneurysms of artery  O033: Percutaneous transluminal stent assisted coil embolisation of single aneurysm of artery  O034: Percutaneous transluminal flow diverting stent assisted coil embolisation of three or more aneurysms of artery  O035: Percutaneous transluminal flow diverting stent assisted coil embolisation of two aneurysms of artery  O036: Percutaneous transluminal flow diverting stent assisted coil embolisation of single aneurysm of artery  O038: Other specified transluminal stent assisted coil embolisation of aneurysm of artery  O039: Unspecified transluminal stent assisted coil embolisation of aneurysm of artery  O041: Percutaneous transluminal liquid polymer embolisation of aneurysm of artery  O042: Percutaneous transluminal stent assisted liquid polymer embolisation of aneurysm of artery  O043: Percutaneous transluminal flow diverting stent embolisation of aneurysm of artery  O048: Other specified other transluminal embolisation of aneurysm of artery  O049: Unspecified other transluminal embolisation of aneurysm of artery |
| Repair of aortic aneurysm rupture or aortic dissection | L181: Emergency replacement of aneurysmal segment of ascending aorta by anastomosis of aorta to aorta  L182: Emergency replacement of aneurysmal segment of thoracic aorta by anastomosis of aorta to aorta NEC  L183: Emergency replacement of aneurysmal segment of suprarenal abdominal aorta by anastomosis of aorta to aorta  L184: Emergency replacement of aneurysmal segment of infrarenal abdominal aorta by anastomosis of aorta to aorta  L185: Emergency replacement of aneurysmal segment of abdominal aorta by anastomosis of aorta to aorta NEC  L186: Emergency replacement of aneurysmal bifurcation of aorta by anastomosis of aorta to iliac artery  L188: Other specified emergency replacement of aneurysmal segment of aorta  L189: Unspecified emergency replacement of aneurysmal segment of aorta  L191: Replacement of aneurysmal segment of ascending aorta by anastomosis of aorta to aorta NEC  L192: Replacement of aneurysmal segment of thoracic aorta by anastomosis of aorta to aorta NEC  L193: Replacement of aneurysmal segment of suprarenal abdominal aorta by anastomosis of aorta to aorta NEC  L194: Replacement of aneurysmal segment of infrarenal abdominal aorta by anastomosis of aorta to aorta NEC  L195: Replacement of aneurysmal segment of abdominal aorta by anastomosis of aorta to aorta NEC  L196: Replacement of aneurysmal bifurcation of aorta by anastomosis of aorta to iliac artery NEC  L198: Other specified other replacement of aneurysmal segment of aorta  L199: Unspecified other replacement of aneurysmal segment of aorta  L265: Percutaneous transluminal insertion of stent into aorta  L266: Transluminal aortic stent graft with fenestration NEC  L267: Transluminal aortic branched stent graft NEC  L254: Operations on aneurysm of aorta NEC  L271: Endovascular insertion of stent graft for infrarenal abdominal aortic aneurysm  L272: Endovascular insertion of stent graft for suprarenal aortic aneurysm  L273: Endovascular insertion of stent graft for thoracic aortic aneurysm  L274: Endovascular insertion of stent graft for aortic dissection in any position  L275: Endovascular insertion of stent graft for aortic aneurysm of bifurcation NEC  L276: Endovascular insertion of stent graft for aorto-uni-iliac aneurysm  L278: Other specified transluminal insertion of stent graft for aneurysmal segment of aorta  L279: Unspecified transluminal insertion of stent graft for aneurysmal segment of aorta  L281: Endovascular insertion of stent for infrarenal abdominal aortic aneurysm  L282: Endovascular insertion of stent for suprarenal aortic aneurysm  L283: Endovascular insertion of stent for thoracic aortic aneurysm  L284: Endovascular insertion of stent for aortic dissection in any position  L285: Endovascular insertion of stent for aortic aneurysm of bifurcation NEC  L286: Endovascular insertion of stent for aorto-uni-iliac aneurysm  L288: Other specified transluminal operations on aneurysmal segment of aorta  L289: Unspecified transluminal operations on aneurysmal segment of aorta  O201: Endovascular placement of one branched stent graft  O202: Endovascular placement of one fenestrated stent graft  O203: Endovascular placement of one stent graft NEC  O204: Endovascular placement of two stent grafts  O205: Endovascular placement of three or more stent grafts  O208: Other specified endovascular placement of stent graft  O209: Unspecified endovascular placement of stent graft |
| Surgical management of acute pancreatitis | J601: Drainage of pancreatic duct  J576: Pancreatic necrosectomy  J602: Open removal of calculus from pancreatic duct  J603: Insertion of T tube into pancreatic duct  J612: Drainage of cyst of pancreas into transposed jejunum  J613: Drainage of cyst of pancreas into jejunum NEC  J614: Drainage of cyst of pancreas NEC |

**Table S2.** ICD-10 codes for covariates

| **Pre-existing Medical Condition(4)** | **Code(5)** | **Timing** |
| --- | --- | --- |
| Diabetes | G590 G632 H280 H360 M142 N083 O240 O241 O243 H350 H352 E103 E113 E123 E133 E143 E10-E14 | Any code from 2003 up to the birth |
| Heart disease - ischaemic | I252 I20 I21 I22 I23 I24 I25 I270 I272 | Any code from 2003 to before the birth episode |
| Heart disease – heart failure and cardiomyopathy | I43 I50 I130 I110 I132 | Any code from 2003 up to the birth |
| Heart disease- congenital | Q20-Q26 | Any code from 2003 up to the birth |
| Heart disease – valve | I34  I35  I05-I08 | Any code from 2003 up to the birth |
| Hypertension | I10-I13 I15 | Any code from 2003 up to the birth |
| Thyroid disease | E035 E038 E039 E050 E051 E052 E055 E058 E059 E062 E063 E065 E069 H062 | Any code from 2003 up to the birth |
| Obstructive lung disorders – cystic fibrosis | E84 | Any code from 2003 up to the birth |
| Restrictive lung disorders | J60 J61 J62 J63 J64 J65 J66 J67 J84 J841 J701 J703 J704 G532  M633 D86 | Any code from 2003 up to the birth |
| Polyarthropothies | I730 J990 M350 M05 M06 M34 L93 M32 | Any code from 2003 up to the birth |
| Obstructive lung disorders | J45 J46 | Any code from 2003 to before the birth episode |
| Inflammatory bowel disease | K51 K50 | Any code from 2003 up to the birth |
| Coeliac | K900 | Any code from 2003 up to the birth |
| Infective Hepatitis | B150 B160 B190 B18 | Any code from 2003 up to the birth |
| Alcohol related liver disease | K701 K702 K703 K704 K70 | Any code from 2003 up to the birth |
| Cirrhosis and liver failure | K717 K740 K741 K742 K744 K745 K746 K72 | Any code from 2003 up to the birth |
| Other liver disease | K743 K754 K711 K762 K763 | Any code from 2003 up to the birth |
| Cerebrovascular disease | I61 I64 I60 I65 I66 I691 I630 I631 I632 I633 I634 I635 I638 I639 I693 G463 G464 G465 G466 G467 G468 I694 I690 G450 G451 G452 G453 G454 G458 G459 G460 G461 G462 | Any code from 2003 up to the birth |
| Epilepsy | G40 G41 | Any code from aged 11+ to before the birth episode |
| Chronic kidney disease | N185 T824 Y602 Y612 Y841 Z491 Z492 Z992 N165 T861 Z940 N183 N184 N187 N188 N189 N186 | Any code from 2003 up to the birth |
| Psoriasis and Eczema | l20 l40 M090 | Any code from aged 11+ up to the birth |
| HIV | F024 B20 B21 B22 B23 B24 R75 Z21 | Any code from 2003 up to the birth |

**Table S2. (Continued)**

| **Pre-existing Medical Condition(4)** | **Code(5)** | **Timing** |
| --- | --- | --- |
| Cancer | C880 C882 C221 C751 C752 C753 C754 C755 C220 C222 C223 C224 C227 C229 C260C261  C268 C269 C300 C301 C380 C381 C382 C383 C384 C388 C390 C398 C399 C750 C758 C759 C883 C884 C887 C889 C797 C795 C784  C785 C793 C794 C787 C780 C781 C783 C788 C790 C791 C792 C796 C798 C799 C786 C782 C81 C91- C95 C82-C85 C86 C90 C40 C41 C70 – C72 C51-C53 C31-C34 C43-C49 C64-C67 C97 C00-C21 C23-C25 C37 C57 C58 C60 C63 C68 C69 C74 C76 C80 C96 C56 C73 C54 C55 C77 C50 D05 | Any code from 2003 up to the birth |
| Transplant | Z94 | Any code from 2003 up to the birth |
| Fibroids | D25 | Any code from 2003 up to the birth |
| Endometriosis | N80 | Any code from 5 years prior to pregnancy up to the birth |
| Inflammatory bowel syndrome | K58 | Any code from 5 years prior to pregnancy up to the birth |
| Post viral and related fatigue | G933 | Any code from 5 years prior to pregnancy up to the birth |
| Fibromyalgia | M797 | Any code from 5 years prior to pregnancy up to the birth |
| Thrombophilia and thromboembolic disease | D68, I74-I76, I26, I80-I82 | Any code from 2003 up to the birth |

| **Substance misuse(6)** | Coded from 5 years prior to index pregnancy up to the birth |
| --- | --- |
| Mental and behavioural disorders due to psychoactive substance use | F11-F17, F19 (not F171) |
| Finding of drugs not normally found in blood | R781-R785 |
| Poisoning by drugs, medicaments and biological substances | T36-T50 (not T506) |
| Poisoning, undetermined intent | Y10-Y14 |
| Drug rehabilitation | Z503 |
| Drug abuse counselling and surveillance | Z715 |
| Drug use | Z722 |
| Personal history of psychoactive substance abuse | Z864 |
| Mental and behavioural disorders due to use of volatile solvents | F18 |
| Accidental poisoning by and exposure to noxious substances | X40–X44, X46-X49 |
| Poisoning by chemical or noxious substance, undetermined intent | Y16-Y19 |
| Special epileptic syndromes - (related to alcohol, drugs, etc) | G405 |

**Table S2. (Continued)**

| **Substance misuse(6)** | Coded from 5 years prior to index pregnancy up to the birth |
| --- | --- |
| Blood-alcohol and blood-drug test | Z040 |
| Alcohol-induced pseudo-Cushing's syndrome | 244 |
| Mental and behavioural disorders due to use of alcohol | F10 |
| Degeneration of nervous system due to alcohol | G312 |
| Alcoholic polyneuropathy | G621 |
| Alcoholic myopathy | G721 |
| Alcoholic cardiomyopathy | I426 |
| Alcoholic gastritis | K292 |
| Alcoholic liver disease | K70 |
| Alcohol-induced acute pancreatitis | K852 |
| Alcohol-induced chronic pancreatitis | K860 |
| Finding of alcohol in blood | R780 |
| Poisoning: antidotes and chelating agents, not elsewhere classified | T506 |
| Toxic effect of alcohol | T51 |
| Accidental poisoning by exposure to alcohol | X45 |
| Poisoning by exposure to alcohol, undetermined intent | Y15 |
| Evidence of alcohol involvement determined by blood alcohol level | Y90 |
| Evidence of alcohol involvement determined by level of intoxication | Y91 |
| Alcohol rehabilitation | Z502 |
| Alcohol abuse counselling and surveillance | Z714 |
| Alcohol use | Z721 |
| **Mental Health conditions/ behavioural disorders(6)** | Coded from 5 years prior to index pregnancy up to the birth |
| Organic, including symptomatic, mental disorders | F00-F09 |
| Schizophrenia, schizotypal and delusional disorders | F20-F29 |
| Mood (affective) disorders | F30-F39 |
| Neurotic, stress-related and somatoform disorders | F40-F48 |
| Behavioural syndromes associated with physiological disturbances and physical factors | F50-F59 |
| Disorders of adult personality and behaviour | F60-F69 |
| Mental retardation | F70-F79 |
| Disorders of psychological development | F80-F89 |
| Behavioural and emotional disorders with onset usually occurring in childhood and adolescence | F90-F98 |
| Unspecified mental disorder | F99 |
| Sedatives, hypnotics and antianxiety drugs | Y47 |
| Psychotropic drugs, not elsewhere classified | Y49 |
| Personal history of other mental and behavioural disorders | Z865 |

**Table S2. (Continued)**

| **Codes for previous pregnancy(7, 8)** | ICD-10: O342, O757, Z354, Z876 and Z875 | Coded for from 2003 up to and including the birth |
| --- | --- | --- |
|  | OPCS-4: R17–R25 | Coded for from 2003 to start of pregnancy |
| **Obesity(8, 9)** | E66 Z684 | Any code from 5 years prior to pregnancy up to and including the birth |

***
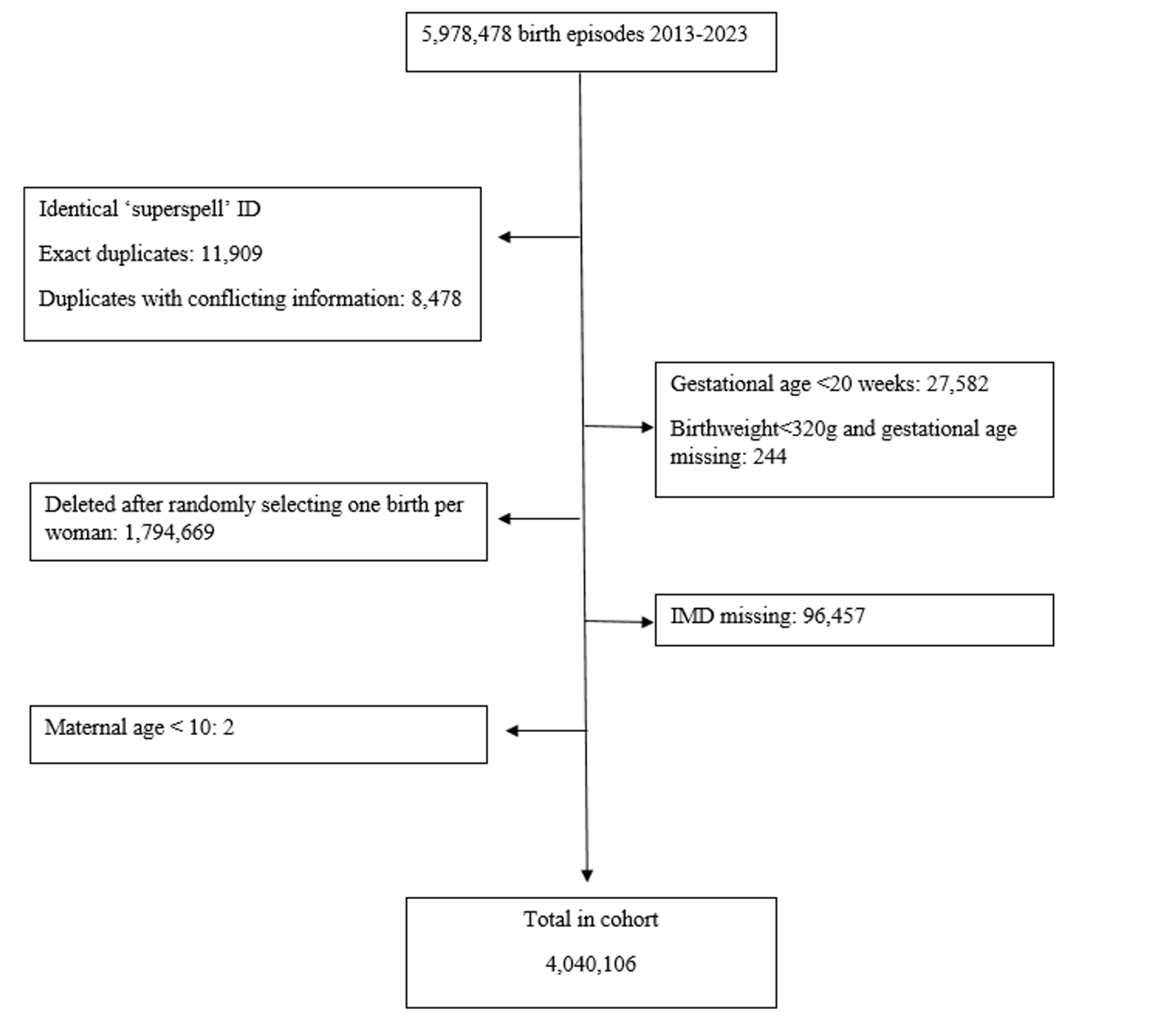
***

**Figure S1.** Flow chart showing the identification of the cohort

**Table S3.** Characteristics of the study population stratified by composite Index of Multiple Deprivation quintile N (%).

|  | **Index of Multiple Deprivation Quintile** | | | | |  |
| --- | --- | --- | --- | --- | --- | --- |
|  | **Most deprived 20%** | **More deprived 20-40%** | **Less deprived 40-60%** | **Less deprived 60-80%** | **Least deprived 80-100%** | **Total** |
| **SMM** |  |  |  |  |  |  |
| No | 1,028,362 (98.1) | 899,119 (98.1) | 765,055 (98.3) | 675,001 (98.4) | 601,151 (98.5) | 3,968,688  (98.2) |
| Yes | 20,025  (1.9) | 17,299  (1.9) | 13,574 (1.7) | 11,227  (1.6) | 9,293  (1.5) | 71,418  (1.8) |
| **Age group** |  |  |  |  |  |  |
| <20 | 55,363  (5.3) | 30,243  (3.3) | 18,850 (2.4) | 12,006  (1.7) | 7,394  (1.2) | 123,856  (3.1) |
| 20-25 | 207,037 (19.7) | 140,181 (15.3) | 95,759 (12.3) | 66,879 (9.7) | 43,695  (7.2) | 553,551  (13.7) |
| 25-30 | 313,768 (29.9) | 262,243 (28.6) | 209,558 (26.9) | 170,878 (24.9) | 130,874 (21.4) | 1,087,321  (26.9) |
| 30-35 | 281,696 (26.9) | 284,168 (31.0) | 263,127 (33.8) | 248,454 (36.2) | 233,664 (38.3) | 1,311,109  (32.5) |
| 35-40 | 149,881 (14.3) | 158,938 (17.3) | 153,684 (19.7) | 150,808 (22.0) | 156,400 (25.6) | 769,711  (19.1) |
| >40 | 40,642  (3.9) | 40,645  (4.4) | 37,651 (4.8) | 37,203 (5.4) | 38,417  (6.3) | 194,558  (4.8) |
| **Ethnicity** |  |  |  |  |  |  |
| White | 665,971 (66.5) | 623,508 (71.7) | 590,306 (80.0) | 554,122 (85.2) | 505,782 (87.2) | 2,939,689  (76.6) |
| Black or Black British - Caribbean | 16,363  (1.6) | 11,260  (1.3) | 5,304  (0.7) | 2,602  (0.4) | 1,517  (0.3) | 37,046  (1.0) |
| Black or Black British - African | 56,529  (5.6) | 33,764  (3.9) | 15,279 (2.1) | 8,403  (1.3) | 5,516  (1.0) | 119,491  (3.1) |
| Asian or Asian British - Indian | 28,995  (2.9) | 39,187  (4.5) | 29,243 (4.0) | 20,346 (3.1) | 17,720  (3.1) | 135,491  (3.5) |
| Asian or Asian British - Pakistani | 76,829 (  7.7) | 37,538  (4.3) | 18,864 (2.6) | 10,219 (1.6) | 6,430  (1.1) | 149,880  (3.9) |
| Asian or Asian British - Bangladeshi | 29,469  (2.9) | 16,301  (1.9) | 6,208  (0.8) | 2,968  (0.5) | 1,700  (0.3) | 56,646  (1.5) |
| Mixed | 24,810  (2.5) | 18,537  (2.1) | 12,763 (1.7) | 10,025 (1.5) | 8,207  (1.4) | 74,342  (1.9) |
| Other | 60,266  (6.0) | 49,783  (5.7) | 34,359 (4.7) | 25,299 (3.9) | 20,908  (3.6) | 190,615  (5.0) |
| Other Asian | 26,605  (2.7) | 28,187  (3.2) | 20,346 (2.8) | 13,833 (2.1) | 10,527  (1.8) | 99,498  (2.6) |
| Other Black | 15,569  (1.6) | 11,312  (1.3) | 5,177  (0.7) | 2,652  (0.4) | 1,748  (0.3) | 36,458  (0.9) |
| **Parity** |  |  |  |  |  |  |
| Multiparous | 659,764 (62.9) | 537,140 (58.6) | 438,060 (56.3) | 382,215 (55.7) | 351,669 (57.6) | 2,368,848  (58.6) |
| Primiparous | 388,623 (37.1) | 379,278 (41.4) | 340,569 (43.7) | 304,013 (44.3) | 258,775 (42.4) | 1,671,258  (41.4) |
| **Rural/Urban** |  |  |  |  |  |  |
| Urban | 1,029,343 (98.2) | 850,301 (92.8) | 637,753 (81.9) | 529,929 (77.2) | 480,662 (78.7) | 3,527,988  (87.3) |
| Rural | 19,044  (1.8) | 66,117  (7.2) | 140,876 (18.1) | 156,299 (22.8) | 129,782 (21.3) | 512,118  (12.7) |

**Table S3. (continued)**

|  | **Index of Multiple Deprivation Quintile** | | | | | |
| --- | --- | --- | --- | --- | --- | --- |
|  | **Most deprived 20%** | **More deprived 20-40%** | **Less deprived 40-60%** | **Less deprived 60-80%** | **Least deprived 80-100%** | **Total** |
| **Neighbourhood % not born in the United Kingdom (UK)** |  |  |  |  |  |  |
| Lowest 20% | 143,288 (13.7) | 135,365 (14.8) | 135,550 (17.4) | 129,127 (18.8) | 90,309 (14.8) | 633,639  (15.7) |
| Lower 20-40% | 130,863 (12.5) | 122,910 (13.4) | 139,272 (17.9) | 151,051 (22.0) | 128,930 (21.1) | 673,026  (16.7) |
| Middle 40-60% | 149,684 (14.3) | 126,040 (13.8) | 144,984 (18.6) | 149,809 (21.8) | 175,144 (28.7) | 745,661  (18.5) |
| Higher 60-80% | 211,775 (20.2) | 176,726 (19.3) | 158,326 (20.3) | 151,993 (22.1) | 168,844 (27.7) | 867,664  (21.5) |
| Highest 80-100% | 412,777 (39.4) | 355,377 (38.8) | 200,497 (25.8) | 104,248 (15.2) | 47,217  (7.7) | 1,120,116 (27.7) |
| **Region** |  |  |  |  |  |  |
| North East | 72,751  (6.9) | 42,623 (4.7) | 23,564 (3.0) | 20,435 (3.0) | 18,176  (3.0) | 177,549  (4.4) |
| North West | 227,447 (21.7) | 104,346 (11.4) | 74,650 (9.6) | 70,271 (10.2) | 54,952  (9.0) | 531,666  (13.2) |
| Yorkshire and Humber | 149,996 (14.3) | 74,309 (8.1) | 60,442 (7.8) | 61,144 (8.9) | 42,613  (7.0) | 388,504  (9.6) |
| East Midlands | 74,323  (7.1) | 69,764 (7.6) | 57,667 (7.4) | 58,831 (8.6) | 57,998  (9.5) | 318,583  (7.9) |
| West Midlands | 170,289 (16.2) | 85,624 (9.3) | 74,546 (9.6) | 59,378 (8.7) | 43,414  (7.1) | 433,251  (10.7) |
| East of England | 56,811  (5.4) | 90,618 (9.9) | 103,793 (13.3) | 94,706 (13.8) | 96,879 (15.9) | 442,807  (11.0) |
| London | 183,610 (17.5) | 260,984 (28.5) | 162,303 (20.8) | 105,007 (15.3) | 56,304  (9.2) | 768,208  (19.0) |
| South East | 64,190  (6.1) | 114,435 (12.5) | 133,841 (17.2) | 137,657 (20.1) | 179,889 (29.5) | 630,012  (15.6) |
| South West | 48,970  (4.7) | 73,715 (8.0) | 87,823 (11.3) | 78,799 (11.5) | 60,219  (9.9) | 349,526  (8.7) |
| **Substance misuse or Smoking** |  |  |  |  |  |  |
| No | 727,128 (69.4) | 683,531 (74.6) | 603,359 (77.5) | 550,446 (80.2) | 510,651 (83.7) | 3,075,115 (76.1) |
| Yes | 321,259 (30.6) | 232,887 (25.4) | 175,270 (22.5) | 135,782 (19.8) | 99,793 (16.3) | 964,991  (23.9) |
| **Pre-existing medical conditions** |  |  |  |  |  |  |
| No | 824,213 (78.6) | 726,596 (79.3) | 616,128 (79.1) | 542,836 (79.1) | 484,860 (79.4) | 3,194,633 (79.1) |
| Yes | 224,174 (21.4) | 189,822 (20.7) | 162,501 (20.9) | 143,392 (20.9) | 125,584 (20.6) | 845,473  (20.9) |
| **Pre-existing mental health problems** |  |  |  |  |  |  |
| No | 906,066 (86.4) | 804,865 (87.8) | 686,335 (88.1) | 607,381 (88.5) | 545,308 (89.3) | 3,549,955 (87.9) |
| Yes | 142,321 (13.6) | 111,553 (12.2) | 92,294 (11.9) | 78,847 (11.5) | 65,136 (10.7) | 490,151  (12.1) |

**Table S3. (continued)**

|  | **Index of Multiple Deprivation Quintile** | | | | | |
| --- | --- | --- | --- | --- | --- | --- |
|  | **Most deprived 20%** | **More deprived 20-40%** | **Less deprived 40-60%** | **Less deprived 60-80%** | **Least deprived 80-100%** | **Total** |
| **Obesity/ Overweight** |  |  |  |  |  |  |
| No | 779,597 (78.5) | 714,653 (81.5) | 622,088 (83.0) | 559,458 (84.3) | 514,193 (86.7) | 3,189,989 (82.3) |
| Yes | 213,613 (21.5) | 161,788 (18.5) | 127,704 (17.0) | 104,319 (15.7) | 78,886 (13.3) | 686,310  (17.7) |
| **Mode of birth** |  |  |  |  |  |  |
| Unassisted vertex birth | 609,611 (58.2) | 505,708 (55.3) | 421,923 (54.3) | 366,773 (53.6) | 323,502 (53.1) | 2,227,517 (55.3) |
| Instrumental birth | 122,666 (11.7) | 124,866 (13.7) | 108,112 (13.9) | 96,084 (14.0) | 85,292 (14.0) | 537,020  (13.3) |
| Breech birth | 4,624  (0.4) | 3,964  (0.4) | 3,502  (0.5) | 2,858  (0.4) | 3,158  (0.5) | 18,106  (0.4) |
| Elective caesarean birth | 124,545 (11.9) | 114,158 (12.5) | 104,524 (13.5) | 99,477 (14.5) | 93,263 (15.3) | 535,967  (13.3) |
| Emergency caesarean birth | 179,325 (17.1) | 159,238 (17.4) | 132,985 (17.1) | 113,814 (16.6) | 97,796 (16.1) | 683,158  (16.9) |
| Other modes of birth | 6,069  (0.6) | 6,680  (0.7) | 5,574  (0.7) | 5,529  (0.8) | 5,821  (1.0) | 29,673  (0.7) |
| **Birth onset method** |  |  |  |  |  |  |
| Spontaneous: the onset of regular contractions whether or not preceded by spontaneous rupture of the membranes | 449,318 (52.0) | 398,247 (53.1) | 339,729 (53.0) | 298,449 (52.8) | 271,827 (53.4) | 1,757,570 (52.8) |
| Any caesarean section carried out immediately following the onset of labour, when the decision was made before labour | 140,541 (16.3) | 120,828 (16.1) | 105,774 (16.5) | 97,175 (17.2) | 91,233 (17.9) | 555,551  (16.7) |
| Surgical induction by amniotomy | 47,538  (5.5) | 40,232 (5.4) | 35,785 (5.6) | 30,227 (5.3) | 27,532  (5.4) | 181,314  (5.4) |
| Medical induction | 159,652 (18.5) | 139,533 (18.6) | 116,190 (18.1) | 101,196 (17.9) | 86,914 (17.1) | 603,485  (18.1) |
| Combination of surgical induction and medical induction | 67,818  (7.8) | 51,134 (6.8) | 43,410 (6.8) | 38,612 (6.8) | 31,077  (6.1) | 232,051  (7.0) |
| **Booked ≥12 weeks** |  |  |  |  |  |  |
| No | 378,100 (71.4) | 362,636 (76.2) | 320,175 (79.3) | 289,469 (81.2) | 270,547 (82.4) | 1,620,927 (77.4) |
| Yes | 151,239 (28.6) | 113,349 (23.8) | 83,758 (20.7) | 67,055 (18.8) | 57,816 (17.6) | 473,217  (22.6) |
| **Total** | **1,048,387 (25.9)** | **916,418 (22.7)** | **778,629 (19.3)** | **686,228 (17.0)** | **610,444 (15.1)** | **4,040,106 (100.0)** |

**Table S4.** Pearson correlation coefficients between the different domains/ subdomains of the Index of Multiple Deprivation

|  | Income | Employment | Crime | Indoor Living Environment | Outdoor Living Environment | Adult Skills | Children and Young People | Wider Barriers | Geographical Barriers |
| --- | --- | --- | --- | --- | --- | --- | --- | --- | --- |
| Income | 1.000 |  |  |  |  |  |  |  |  |
| Employment | 0.901 | 1.000 |  |  |  |  |  |  |  |
| Crime | 0.627 | 0.577 | 1.000 |  |  |  |  |  |  |
| Indoor Living Environment | 0.278 | 0.248 | 0.273 | 1.000 |  |  |  |  |  |
| Outdoor Living Environment | 0.334 | 0.226 | 0.476 | 0.279 | 1.000 |  |  |  |  |
| Adult Skills | 0.751 | 0.768 | 0.462 | 0.115 | 0.120 | 1.000 |  |  |  |
| Children and Young People | 0.712 | 0.705 | 0.459 | 0.184 | 0.074 | 0.746 | 1.000 |  |  |
| Wider Barriers | 0.471 | 0.317 | 0.443 | 0.271 | 0.566 | 0.191 | 0.206 | 1.000 |  |
| Geographical Barriers | -0.421 | -0.357 | -0.428 | -0.319 | -0.456 | -0.218 | -0.210 | -0.443 | 1.000 |


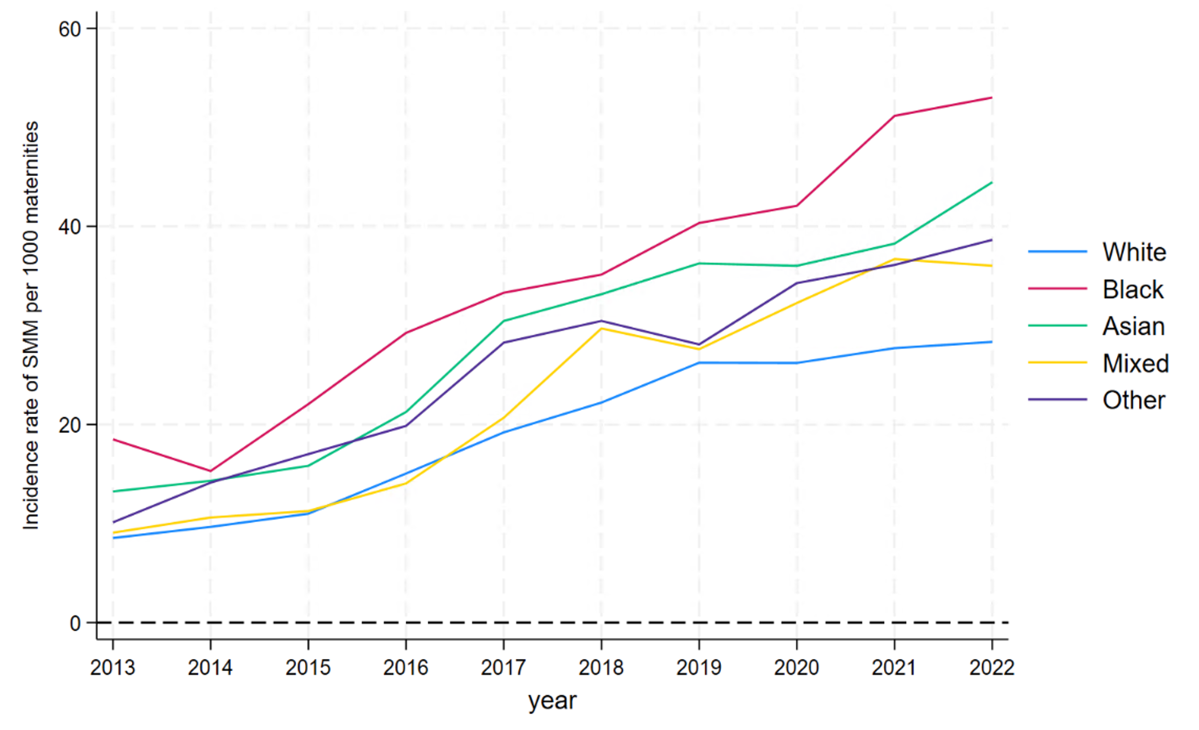

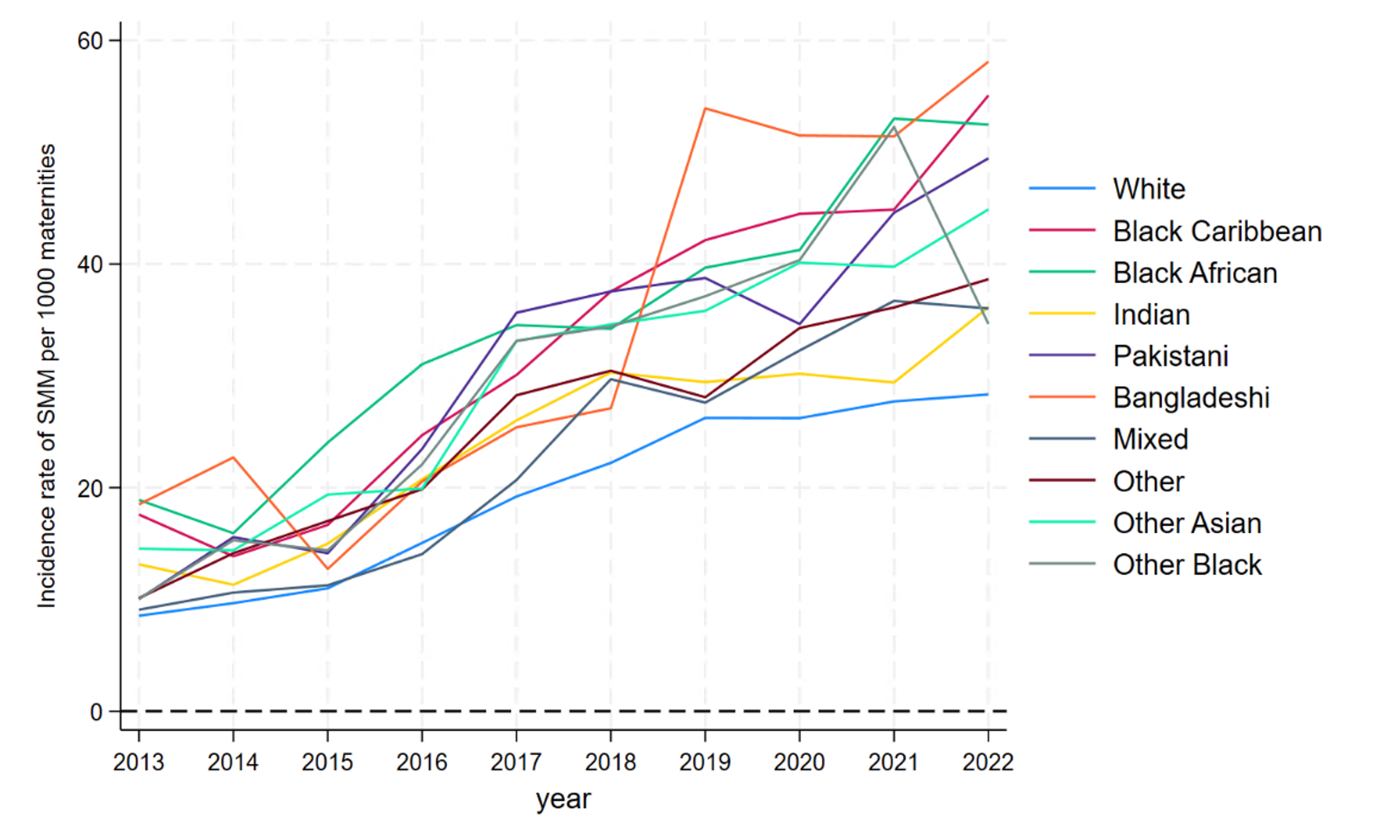


**Figure S3.** The crude incidence rate (N per 1000 maternities) of severe maternal morbidity (SMM) over time by ten ethnic groups

**Figure S2.** The crude incidence rate (N per 1000 maternities) of severe maternal morbidity (SMM) over time by five ethnic groups

**Figure S4.** The proportion (%) of each severe maternal morbidity (SMM) diagnoses (ICD-10) or procedures (OPCS-4) in the final composite outcome

**Table S5**. Univariable and multivariable analysis showing risk ratios and their 95% confidence intervals (RR (95% CI)) between the composite Index of Multiple Deprivation (IMD) quintiles and severe maternal morbidity (SMM) compared to the least deprived IMD quintile. There is no Model 3 as Model 1 did not require any additional adjustments based on the DAG *Model 1: Adjusted for year, age, parity and ethnicity. **Model 2: Model 1 additionally adjusted for substance misuse/pre-existing medical conditions/pre-existing mental health conditions. ICC = Intra-class Correlation Coefficient.

|  | **Unadjusted** | **Model 1***  **(final model)** | **Model 2**** |
| --- | --- | --- | --- |
| Composite Index of Multiple Deprivation quintile | Risk Ratio  (95% CI) | Risk Ratio  (95% CI) | Risk Ratio  (95% CI) |
| Most deprived 20% | 1.24 | 1.21 | 1.19 |
|  | [1.20 1.27] | [1.17 1.24] | [1.16 1.22] |
| More deprived 20- 40% | 1.23 | 1.18 | 1.17 |
|  | [1.20 1.27] | [1.15 1.21] | [1.14 1.20] |
| Less deprived 40-60% | 1.15 | 1.11 | 1.10 |
|  | [1.11 1.18] | [1.08 1.14] | [1.07 1.14] |
| Less deprived 60-80% | 1.07 | 1.05 | 1.05 |
|  | [1.04 1.11] | [1.02 1.09] | [1.02 1.08] |
| Least deprived 80-100% | 1[ref] | 1[ref] | 1[ref] |
| ICC | 0.036  (0.033-0.038) | 0.027  (0.025-0.030) | 0.027  (0.025-0.030) |

**Table S6.** Univariable and multivariable analysis showing risk ratios and their 95% confidence intervals (RR (95% CI)) between income quintiles and severe maternal morbidity (SMM) compared to the least deprived income quintile. *Model 1: Adjusted for year, age, parity and ethnicity **Model 2: Model 1 additionally adjusted for % neighbourhood country of birth/neighbourhood English language proficiency ***Model 3: Model 2 additionally adjusted for substance misuse/pre-existing medical conditions/pre-existing mental health conditions. ICC = Intra-class Correlation Coefficient

|  | **Unadjusted** | **Model 1*** | **Model 2****  **(final model)** | **Model 3***** |
| --- | --- | --- | --- | --- |
| Income quintile | Risk Ratio  (95% CI) | Risk Ratio  (95% CI) | Risk Ratio  (95% CI) | Risk Ratio  (95% CI) |
| Most deprived 20% | 1.23 | 1.21 | 1.16 | 1.14 |
|  | [1.19 1.26] | [1.18 1.24] | [1.12 1.20] | [1.10 1.17] |
| More deprived 20-40% | 1.19 | 1.16 | 1.12 | 1.10 |
|  | [1.16 1.22] | [1.13 1.19] | [1.08 1.15] | [1.06 1.13] |
| Less deprived 40-60% | 1.12 | 1.10 | 1.07 | 1.06 |
|  | [1.09 1.16] | [1.07 1.13] | [1.04 1.10] | [1.03 1.10] |
| Less deprived 60-80% | 1.04 | 1.04 | 1.04 | 1.03 |
|  | [1.01 1.08] | [1.01 1.08] | [1.00 1.07] | [1.00 1.06] |
| Least deprived 80-100% | 1[ref] | 1[ref] | 1[ref] | 1[ref] |
| ICC | 0.035  (0.033-0.038) | 0.028  (0.025-0.030) | 0.027  (0.025-0.030) | 0.027  (0.025-0.029) |

**Table S7.** Univariable and multivariable analysis showing risk ratios and their 95% confidence intervals (RR (95% CI)) between employment quintiles and severe maternal morbidity (SMM) compared to the least deprived employment quintiles. *Model 1: Adjusted for year, age, parity and ethnicity **Model 2: Model 1 additionally adjusted for %neighbourhood country of birth/neighbourhood English language proficiency ***Model 3: Model 2 additionally adjusted for substance misuse/pre-existing medical conditions/pre-existing mental health conditions. ICC = Intra-class Correlation Coefficient

|  | **Unadjusted** | **Model 1*** | **Model 2****  **(final model)** | **Model 3***** |
| --- | --- | --- | --- | --- |
| Employment quintile | Risk Ratio  (95% CI) | Risk Ratio  (95% CI) | Risk Ratio  (95% CI) | Risk Ratio  (95% CI) |
| Most deprived 20% | 1.15 | 1.16 | 1.15 | 1.12 |
|  | [1.11 1.18] | [1.13 1.20] | [1.11 1.19] | [1.09 1.16] |
| More deprived 20-40% | 1.15 | 1.15 | 1.13 | 1.11 |
|  | [1.12 1.18] | [1.11 1.18] | [1.09 1.16] | [1.07 1.14] |
| Less deprived 40-60% | 1.11 | 1.10 | 1.08 | 1.07 |
|  | [1.08 1.15] | [1.07 1.13] | [1.05 1.11] | [1.04 1.10] |
| Less deprived 60-80% | 1.07 | 1.06 | 1.08 | 1.07 |
|  | [1.03 1.10] | [1.03 1.09] | [1.04 1.11] | [1.04 1.10] |
| Least deprived 80-100% | 1[ref] | 1[ref] | 1[ref] | 1[ref] |
| ICC | 0.036  (0.034-0.039) | 0.028  (0.026-0.030) | 0.027  (0.025-0.030) | 0.027  (0.025-0.30) |

**Table S8.** Univariable and multivariable analysis showing risk ratios and their 95% confidence intervals (RR (95% CI)) between indoor living environment quintiles and severe maternal morbidity (SMM) compared to the least deprived indoor living environment quintile. *Model 1: Adjusted for year, age, parity and ethnicity **Model 2: Model 1 additionally adjusted for neighbourhood income and English language proficiency ***Model 3: Model 2 additionally adjusted for substance misuse/pre-existing mental health conditions. ICC = Intra-class Correlation Coefficient

|  | **Unadjusted** | **Model 1*** | **Model 2****  **(final model)** | **Model 3***** |
| --- | --- | --- | --- | --- |
| Indoors Living Environment quintile | Risk Ratio  (95% CI) | Risk Ratio  (95% CI) | Risk Ratio  (95% CI) | Risk Ratio  (95% CI) |
| Most deprived 20% | 1.10 | 1.09 | 1.05 | 1.04 |
|  | [1.07 1.13] | [1.06 1.12] | [1.02 1.08] | [1.03 1.07] |
| More deprived 20-40% | 1.12 | 1.08 | 1.05 | 1.05 |
|  | [1.09 1.15] | [1.06 1.11] | [1.02 1.08] | [1.02 1.07] |
| Less deprived 40-60% | 1.10 | 1.06 | 1.04 | 1.02 |
|  | [1.07 1.13] | [1.04 1.09] | [1.01 1.07] | [0.99 1.05] |
| Less deprived 60-80% | 1.07 | 1.05 | 1.03 | 1.03 |
|  | [1.04 1.10] | [1.02 1.07] | [1.00 1.06] | [1.00 1.05] |
| Least deprived 80-100% | 1[ref] | 1[ref] | 1[ref] | 1[ref] |
| ICC | 0.037  (0.035-0.040) | 0.028  (0.026-0.031) | 0.028  (0.025-0.030) | 0.028  (0.025-0.030) |

**Table S9.** Univariable and multivariable analysis showing risk ratios and their 95% confidence intervals (RR (95% CI)) between outdoor living environment quintiles and severe maternal morbidity (SMM) compared to the least deprived outdoor living environment quintile. *Model 1: Adjusted for year, age, parity and ethnicity **Model 2: Model 1 additionally adjusted for neighbourhood country of birth/ neighbourhood English language proficiency and neighbourhood income ***Model 3: Model 2 additionally adjusted for substance misuse pre-existing mental health conditions. ICC = Intra-class Correlation Coefficient

|  | **Unadjusted** | **Model 1*** | **Model 2****  **(final model)** | **Model 3***** |
| --- | --- | --- | --- | --- |
| Outdoor Living Environment quintile | Risk Ratio  (95% CI) | Risk Ratio  (95% CI) | Risk Ratio  (95% CI) | Risk Ratio  (95% CI) |
| Most deprived 20% | 1.38 | 1.20 | 1.06 | 1.06 |
|  | [1.34 1.42] | [1.16 1.23] | [1.03 1.10] | [1.03 1.10] |
| More deprived 20-40% | 1.17 | 1.10 | 1.03 | 1.02 |
|  | [1.14 1.20] | [1.07 1.14] | [1.00 1.06] | [1.00 1.05] |
| Less deprived 40-60% | 1.08 | 1.06 | 1.02 | 1.01 |
|  | [1.05 1.11] | [1.03 1.09] | [0.99 1.05] | [0.98 1.04] |
| Less deprived 60-80% | 1.03 | 1.02 | 1.00 | 1.00 |
|  | [1.00 1.06] | [0.99 1.05] | [0.97 1.03] | [0.97 1.03] |
| Least deprived 80-100% | 1[ref] | 1[ref] | 1[ref] | 1[ref] |
| ICC | 0.031  (0.029-0.034) | 0.027  (0.025-0.029) | 0.027  (0.024-0.029) | 0.027  (0.024-0.029) |

**Table S10.** Univariable and multivariable analysis showing risk ratios and their 95% confidence intervals (RR (95% CI)) between adult skills quintiles and severe maternal morbidity (SMM) compared to the least deprived adult skills quintile. There is no Model 1 as Model 2 did not require any additional adjustments based on the DAG *Model 2: Adjusted for year, age, parity and ethnicity. **Model 3: Model 2 additionally adjusted for substance misuse/pre-existing medical conditions/pre-existing mental health conditions. ICC = Intra-class Correlation Coefficient

|  | **Unadjusted** | **Model 2***  **(final model)** | **Model 3**** |
| --- | --- | --- | --- |
| Adult Skills quintile | Risk Ratio  (95% CI) | Risk Ratio  (95% CI) | Risk Ratio  (95% CI) |
| Most deprived 20% | 1.02 | 1.10 | 1.07 |
|  | [0.99 1.05] | [1.06 1.13] | [1.04 1.10] |
| More deprived 20-40% | 1.04 | 1.09 | 1.08 |
|  | [1.01 1.07] | [1.06 1.12] | [1.05 1.11] |
| Less deprived 40-60% | 1.00 | 1.05 | 1.04 |
|  | [0.97 1.03] | [1.02 1.08] | [1.01 1.07] |
| Less deprived 60-80% | 0.97 | 1.02 | 1.01 |
|  | [0.95 1.00] | [0.99 1.05] | [0.98 1.04] |
| Least deprived 80-100% | 1[ref] | 1[ref] | 1[ref] |
| ICC | 0.037  (0.035-0.040) | 0.028  (0.026-0.031) | 0.028  (0.026-0.031) |

**Table S11.** Univariable and multivariable analysis showing risk ratios and their 95% confidence intervals (RR (95% CI)) between children and young people quintiles and severe maternal morbidity (SMM) compared to the least deprived children and young people quintile. *Model 1: Adjusted for year, age, parity and ethnicity **Model 2: Model 1 additionally adjusted for neighbourhood country of birth/neighbourhood English language proficiency. ICC = Intra-class Correlation Coefficient

|  | **Unadjusted** | **Model 1*** | **Model 2**  **(final model)**** |
| --- | --- | --- | --- |
| Children and Young People quintile | Risk Ratio  (95% CI) | Risk Ratio  (95% CI) | Risk Ratio  (95% CI) |
| Most deprived 20% | 1.00 | 1.07 | 1.06 |
|  | [0.97 1.02] | [1.04 1.10] | [1.03 1.09] |
| More deprived 20-40% | 1.06 | 1.09 | 1.07 |
|  | [1.03 1.09] | [1.06 1.12] | [1.04 1.10] |
| Less deprived 40-60% | 1.08 | 1.07 | 1.04 |
|  | [1.05 1.11] | [1.04 1.10] | [1.02 1.07] |
| Less deprived 60-80% | 1.06 | 1.05 | 1.04 |
|  | [1.03 1.09] | [1.02 1.08] | [1.01 1.07] |
| Least deprived 80-100% | 1[ref] | 1[ref] | 1[ref] |
| ICC | 0.037  (0.034-0.039) | 0.029  (0.026-031) | 0.028  (0.025-0.030) |

**Table S12.** Univariable and multivariable analysis showing risk ratios and their 95% confidence intervals (RR (95% CI)) between wider barriers quintiles and severe maternal morbidity (SMM) compared to the least deprived wider barriers quintile. *Model 1: Adjusted for year, age, parity and ethnicity **Model 2: Model 1 + additionally adjusted for neighbourhood country of birth/neighbourhood income/neighbourhood English language proficiency and urban/rural ***Model 3: Model 2 additionally adjusted for substance misuse/ pre-existing medical conditions/pre-existing mental health conditions. ICC = Intra-class Correlation Coefficient

|  | **Unadjusted** | **Model 1*** | **Model 2****  **(final model)** | **Model 3***** |
| --- | --- | --- | --- | --- |
| Wide Barriers quintile | Risk Ratio  (95% CI) | Risk Ratio  (95% CI) | Risk Ratio  (95% CI) | Risk Ratio  (95% CI) |
| Most deprived 20% | 1.49 | 1.26 | 1.14 | 1.14 |
|  | [1.45 1.53] | [1.23 1.30] | [1.10 1.20] | [1.10 1.20] |
| More deprived 20-40% | 1.14 | 1.08 | 1.01 | 1.01 |
|  | [1.11 1.17] | [1.05 1.11] | [0.98 1.05] | [0.98 1.05] |
| Less deprived 40-60% | 1.07 | 1.05 | 1.01 | 1.01 |
|  | [1.04 1.10] | [1.02 1.08] | [0.98 1.05] | [0.97 1.05] |
| Less deprived 60-80% | 1.03 | 1.03 | 1.01 | 1.01 |
|  | [1.00 1.06] | [1.00 1.06] | [0.98 1.04] | [0.98 1.04] |
| Least deprived 80-100% | 1[ref] | 1[ref] | 1[ref] | 1[ref] |
| ICC | 0.030  (0.028-0.032) | 0.027  (0.025-0.029) | 0.026  (0.024-0.029) | 0.026  (0.024-0.029) |

**Table S13.** Univariable and multivariable analysis showing risk ratios and their 95% confidence intervals (RR (95% CI)) between geographical barriers quintiles and severe maternal morbidity (SMM) compared to the least deprived geographical barriers quintile. *Model 1: Adjusted for year, age, parity and ethnicity **Model 2: Model 1 + additionally adjusted for neighbourhood country of birth/neighbourhood income, air pollution and urban/rural ***Model 3: Model 2 additionally adjusted for substance misuse/pre-existing mental health conditions. ICC = Intra-class Correlation Coefficient

|  | **Unadjusted** | **Model 1*** | **Model 2****  **(final model)** | **Model 3***** |
| --- | --- | --- | --- | --- |
| Geographical Barriers quintile | Risk Ratio  (95% CI) | Risk Ratio  (95% CI) | Risk Ratio  (95% CI) | Risk Ratio  (95% CI) |
| Most deprived 20% | 0.73 | 0.83 | 0.92 | 0.92 |
|  | [0.71 0.75] | [0.81 0.85] | [0.89 0.95] | [0.89 0.95] |
| More deprived 20-40% | 0.78 | 0.87 | 0.94 | 0.93 |
|  | [0.76 0.80] | [0.85 0.89] | [0.91 0.96] | [0.90 0.95] |
| Less deprived 40-60% | 0.83 | 0.91 | 0.96 | 0.96 |
|  | [0.81 0.86] | [0.89 0.94] | [0.94 0.99] | [0.94 1.00] |
| Less deprived 60-80% | 0.87 | 0.92 | 0.95 | 0.95 |
|  | [0.85 0.89] | [0.90 0.94] | [0.93 0.97] | [0.93 0.98] |
| Least deprived 80-100% | 1[ref] | 1[ref] | 1[ref] | 1[ref] |
| ICC | 0.039  (0.036-0.043) | 0.028  (0.025-0.030) | 0.027  (0.025-0.029) | 0.027  (0.025-0.029) |

**Table S14.** Univariable and multivariable analysis showing risk ratios and their 95% confidence intervals (RR (95% CI)) between crime quintiles and severe maternal morbidity (SMM) compared to the least deprived crime quintile. *Model 1: Adjusted for year, age, parity and ethnicity **Model 2: Model 1 + additionally adjusted for neighbourhood country of birth/neighbourhood income/ English language proficiency and urban/rural ***Model 3: Model 2 Additionally adjusted for substance misuse/pre-existing medical and mental health conditions. ICC = Intra-class Correlation Coefficient

|  | **Unadjusted** | **Model 1*** | **Model 2****  **(final model)** | **Model 3***** |
| --- | --- | --- | --- | --- |
| Crime quintile | Risk Ratio  (95% CI) | Risk Ratio  (95% CI) | Risk Ratio  (95% CI) | Risk Ratio  (95% CI) |
| Most deprived 20% | 1.23 | 1.14 | 0.98 | 0.98 |
|  | [1.19 1.26] | [1.11 1.17] | [0.95 1.02] | [0.94 1.01] |
| More deprived 20-40% | 1.21 | 1.13 | 1.00 | 0.99 |
|  | [1.17 1.24] | [1.10 1.16] | [0.97 1.03] | [0.96 1.03] |
| Less deprived 40-60% | 1.14 | 1.08 | 0.99 | 0.99 |
|  | [1.11 1.18] | [1.05 1.11] | [0.96 1.02] | [0.96 1.02] |
| Less deprived 60-80% | 1.05 | 1.03 | 0.98 | 0.98 |
|  | [1.02 1.09] | [1.00 1.06] | [0.95 1.01] | [0.95 1.01] |
| Least deprived 80-100% | 1[ref] | 1[ref] | 1[ref] | 1[ref] |
| ICC | 0.035  (0.033-0.038) | 0.028  (0.026-0.031) | 0.027  (0.025-0.030) | 0.027  (0.025-0.030) |

**Figure S5.** Adjusted risk ratio and their 95% confidence intervals (RR (95% CI) of conditions which make up the largest proportion of ‘all other SMM’, in each of the composite Index of Multiple Deprivation (IMD) quintiles compared to the least deprived quintile. 1= least deprived quintile, 5= most deprived quintile

* *Adjusted for age, year, parity and ethnicity*

**Figure S6.** The average adjusted predictive margins and their 95% confidence intervals (95% CI) of severe maternal morbidity (SMM) for women living in each adult skills quintile stratified by ethnicity


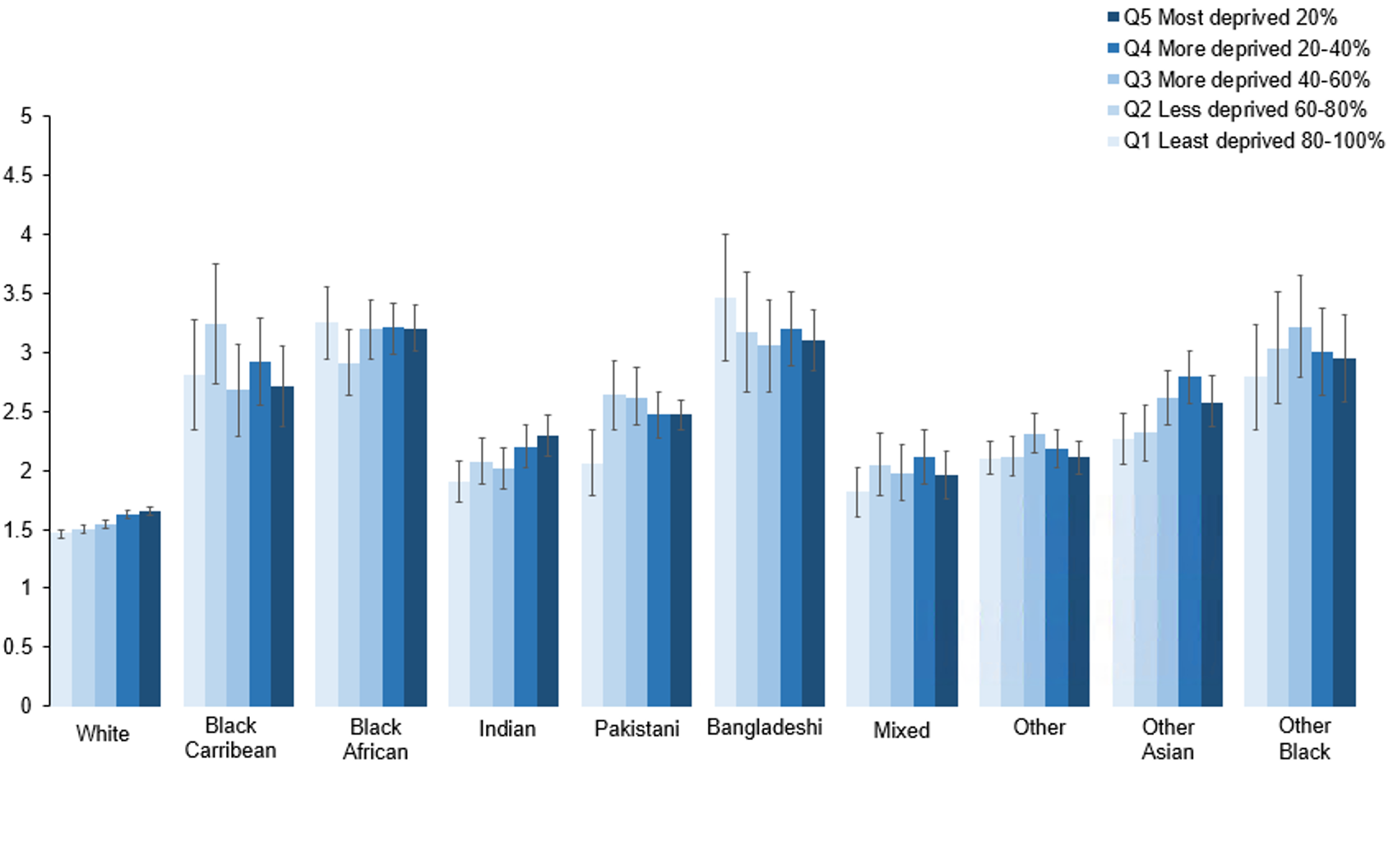


P_interaction_ = 0.048

Probability (%) of SMM (95% CI)


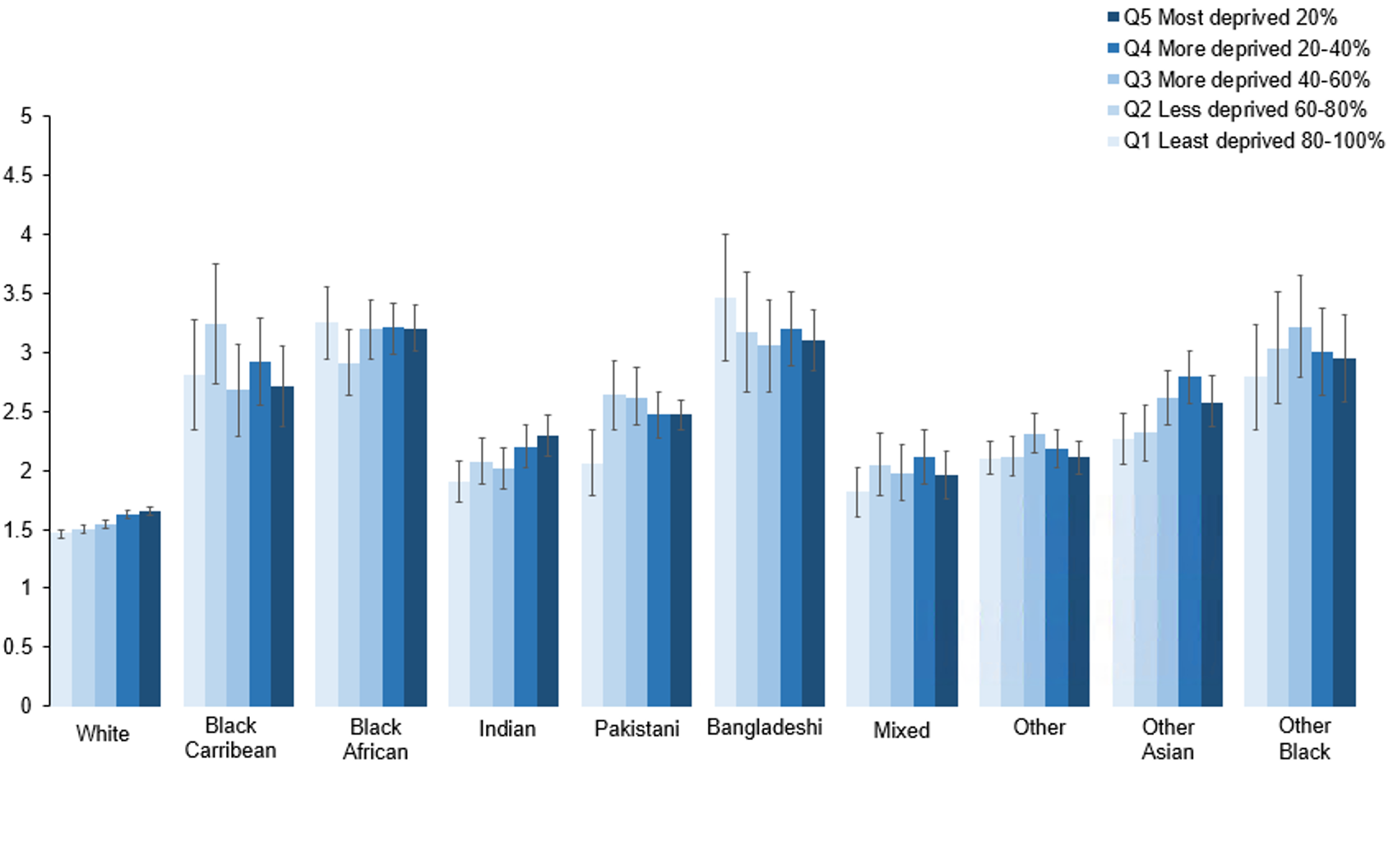
**
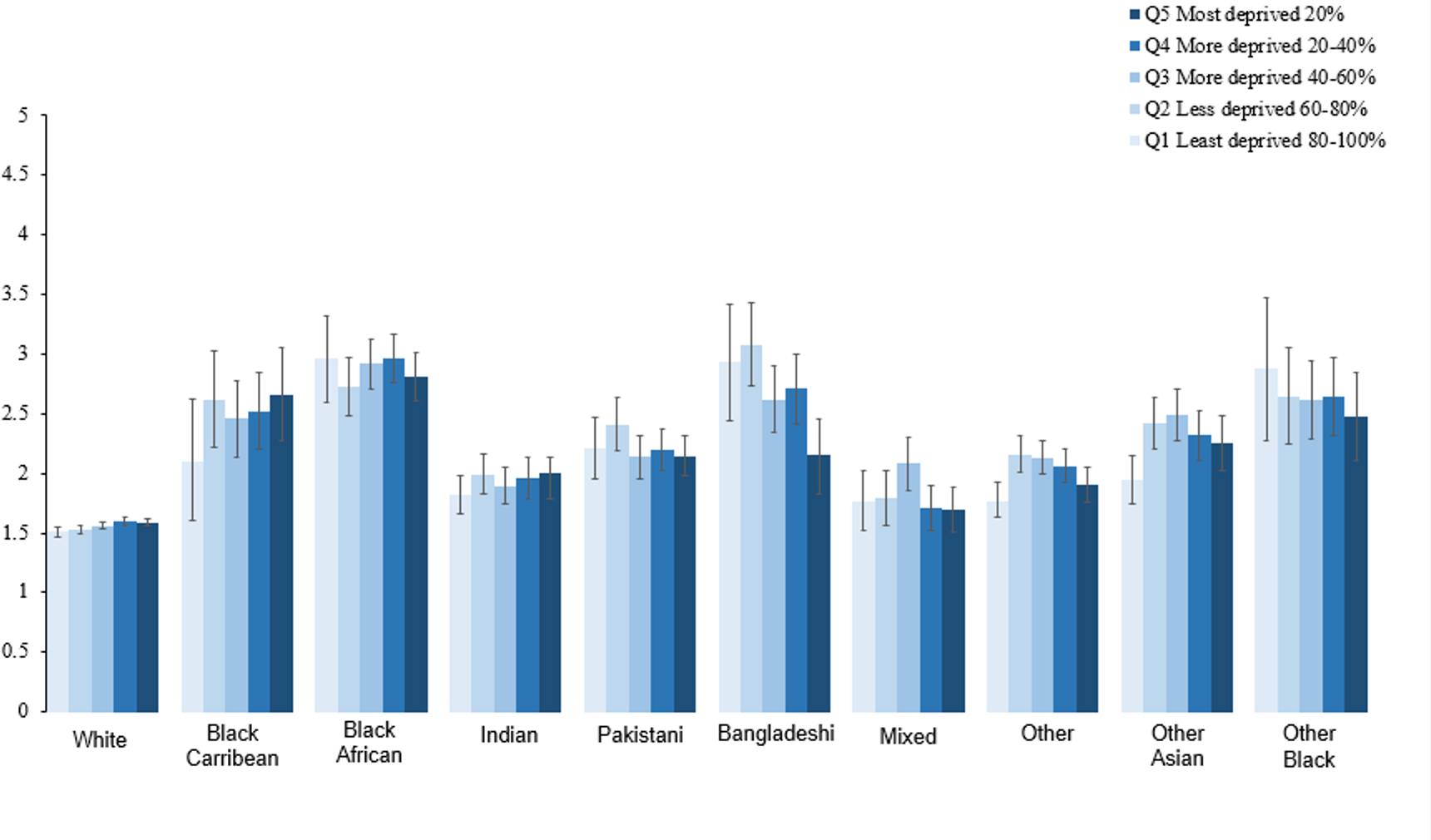
Figure S7.** The average adjusted predictive margins and their 95% confidence intervals (95% CI) of severe maternal morbidity (SMM) for women living in each children and young people quintile stratified by ethnicity

P_interaction_ = 0.01

Probability (%) of SMM (95% CI)

References

1. The Area Based Analysis Unit OfNS. Understanding patterns of deprivation. Regional Trends. 2009;41(1):93-114.

2. McLennan D NS, Noble M, Plunkett E, Wright G, Gutacker N. English indices of deprivation 2019: technical report. London: Ministry of Housing, Communities & Local Government; 2019. Available from: <https://assets.publishing.service.gov.uk/media/5d8b387740f0b609909b5908/IoD2019_Technical_Report.pdf>.

3. Statistics OfN. Understanding consistency of ethnicity data recorded in health-related administrative datasets in England: 2011 to 2021.

4. D’Arcy RS. Investigating the Health and Care Needs of Pregnant Women with Multiple Long-Term Conditions [PhD thesis (awaiting publication)]2024.

5. Lee SI, Azcoaga-Lorenzo A, Agrawal U, Kennedy JI, Fagbamigbe AF, Hope H, et al. Epidemiology of pre-existing multimorbidity in pregnant women in the UK in 2018: a population-based cross-sectional study. BMC pregnancy and childbirth. 2022;22(1):120.

6. Harron K, Gilbert R, Fagg J, Guttmann A, van der Meulen J. Associations between pre-pregnancy psychosocial risk factors and infant outcomes: a population-based cohort study in England. The Lancet Public Health. 2021;6(2):e97-e105.

7. Cromwell DA, Knight HE, Gurol-Urganci I. Parity derived for pregnant women using historical administrative hospital data: accuracy varied among patient groups. Journal of clinical epidemiology. 2014;67(5):578-85.

8. Sandall J, Murrells T, Dodwell M, Gibson R, Bewley S, Coxon K, et al. The efficient use of the maternity workforce and the implications for safety and quality in maternity care: a population-based, cross-sectional study. Health Services and Delivery Research. 2014;2(38).

9. Jardine J, Blotkamp A, Gurol-Urganci I, Knight H, Harris T, Hawdon J, et al. Risk of complicated birth at term in nulliparous and multiparous women using routinely collected maternity data in England: cohort study. Bmj. 2020;371.
